# Using antisense oligonucleotides for the physiological modulation of the alternative splicing of *NF1* exon 23a during PC12 neuronal differentiation

**DOI:** 10.1038/s41598-021-83152-w

**Published:** 2021-02-11

**Authors:** Josep Biayna, Helena Mazuelas, Bernat Gel, Ernest Terribas, Gabrijela Dumbovic, Inma Rosas, Juana Fernández-Rodriguez, Ignacio Blanco, Elisabeth Castellanos, Meritxell Carrió, Conxi Lazaro, Eduard Serra

**Affiliations:** 1 Hereditary Cancer Group, Germans Trias i Pujol Research Institute (IGTP), Can Ruti Biomedical Campus, Badalona, Barcelona, Spain; 2 BioFrontiers Institute, University of Colorado Boulder, Boulder, CO USA; 3 Hereditary Cancer Program, Catalan Institute of Oncology (ICO), Institut d’Investigació Biomédica de Bellvitge (IDIBELL), Hospitalet de Llobregat, Barcelona, Spain; 4 Centro de Investigación Biomédica en Red de Cáncer (CIBERONC), Madrid, Spain; 5 Clinical Genetics and Genetic Counseling Program, Germans Trias i Pujol Hospital, Can Ruti Biomedical Campus, Badalona, Barcelona Spain; 6 Present Address: Institute for Research in Biomedicine (IRB Barcelona), Parc Cientific de Barcelona, Barcelona, Spain

**Keywords:** RNA splicing, Cell signalling

## Abstract

Neurofibromatosis Type 1 (NF1) is a genetic condition affecting approximately 1:3500 persons worldwide. The *NF1* gene codes for neurofibromin protein, a GTPase activating protein (GAP) and a negative regulator of RAS. The *NF1* gene undergoes alternative splicing of exon 23a (E23a) that codes for 21 amino acids placed at the center of the GAP related domain (GRD). E23a-containing type II neurofibromin exhibits a weaker Ras-GAP activity compared to E23a-less type I isoform. Exon E23a has been related with the cognitive impairment present in NF1 individuals. We designed antisense Phosphorodiamidate Morpholino Oligomers (PMOs) to modulate E23a alternative splicing at physiological conditions of gene expression and tested their impact during PC12 cell line neuronal differentiation. Results show that any dynamic modification of the natural ratio between type I and type II isoforms disturbed neuronal differentiation, altering the proper formation of neurites and deregulating both the MAPK/ERK and cAMP/PKA signaling pathways. Our results suggest an opposite regulation of these pathways by neurofibromin and the possible existence of a feedback loop sensing neurofibromin-related signaling. The present work illustrates the utility of PMOs to study alternative splicing that could be applied to other alternatively spliced genes in vitro and in vivo.

## Introduction

Neurofibromatosis type 1 (NF1) is an autosomal dominant inherited neurocutaneous disease affecting about 1:3500 persons worldwide, caused by mutations in the *NF1* gene^1^. The most frequent clinical manifestations of NF1 are the presence of skin *cafe au lait* macules (CALMs), skin-fold freckling, iris hamartomas termed Lisch nodules and the development of multiple cutaneous neurofibromas, benign tumors originating in the peripheral nervous system (PNS). The presence of cognitive impairment and a high predisposition to develop other peripheral and central nervous system (CNS) tumors are also common and important clinical manifestations^1^.

The *NF1* gene is located in the pericentromeric region of chromosome 17, at 17q11.2, and spans about 280 Kb of genomic DNA^2^. The gene is composed of 60 exons, 57 are constitutive exons and 3 are tissue-specific alternatively spliced exons (9a, 23a and 48a)^3–5^. This gene encodes for a protein termed neurofibromin. In its central region, neurofibromin contains a small domain (GAP-related domain, GRD) with a high sequence similarity to the family of GTPase activating proteins of Ras (Ras-GAPs)^6–8^. Neurofibromin acts as a negative regulator of Ras signaling by enhancing the intrinsic capacity of Ras to hydrolyze a phosphate of the Ras-bound GTP, turning this active form into an inactive Ras-GDP^9^. Activated Ras proteins interact with several downstream effectors, regulating different signaling pathways, like the MAPK/ERK pathway, and cellular processes such as cell proliferation and differentiation^10^.

Alternative splicing of exon 23a (E23a) of the *NF1* gene generates two isoforms named type I and II. Splicing factors regulating this alternative splicing have been investigated^11–13^. E23a lies within the GRD coding sequence and is constituted by 63 bp that encodes for 21 amino acids placed at the center of the GRD domain^5^. Type II transcript contains E23a and exhibits a ten-fold lower Ras-GAP activity compared to E23a-less type I isoform^14–16^. Thus, type II isoform is a weaker negative regulator of Ras than type I. Alternative splicing is a major source of proteome diversity and a key regulator of physiological processes, like neuronal branching and differentiation, brain development, etc^17,18^. However, the physiological relevance of alternative splicing is still largely unknown. Likewise, the impact on cell physiology of the expression and regulation of *NF1* type I/II isoforms is still not completely understood. Many studies have characterized the expression of both isoforms in different tissues and organs (even in different organ parts), developmental stages and in tumors^5,19–27^. While type II isoform is predominantly expressed in most adult tissues, type I isoform has been found expressed in neurons of the CNS at certain developmental stages and in adulthood^5,19,23–25,27^. The switch from type II to type I has also been characterized upon the induction of neural differentiation of PC12 cells by nerve growth factor^28^. The increase in GAP activity due to the higher expression of neurofibromin type I isoform correlates with a down-regulation of Ras activity during PC12 neurite elongation^15^, and also in embryonic stem cell-derived neurons^29^. Neurofibromin has also been shown to regulate cAMP-dependent signaling pathways^30,31^ and PC12 neuronal differentiation is in fact dependent on the interplay of both the MAPK/ERK signaling and the cAMP/PKA signaling^32–34^. Finally, E23a has also been studied in relation to NF1 clinical manifestations. Genetically engineered mouse models either lacking^35^ or retaining E23a^36^ recapitulate NF1 learning and cognitive disabilities. NF1-dependent activation of cAMP/PKA signaling has also been demonstrated to be essential for mediating *Drosophila* learning and memory^37^.

Antisense oligonucleotides (ASOs) have been used to inhibit gene expression and to modify splicing outcomes^38–41^. For instance, ASOs have been successfully used to totally or partially overcome the impact of nonsense, frame-shift and deep intronic mutations at the expression level^42–44^. However, the use of ASOs to modulate alternatively spliced exons has almost not been explored^45–47^. Among different types of ASOs, Phosphorodiamidate Morpholino Oligomers (PMOs) have highly desirable molecular properties that make them suitable for splicing modulation. In PMOs, the deoxyribose sugar is replaced with a six-member morpholino ring, and the phosphodiester inter-subunit bonds are replaced with phosphorodiamidate linkages, a structure that provides a high-targeting specificity, stability, low toxicity and resistance to nucleases^48^, assuring the maintenance of a good long-term activity within the cell. PMOs can be delivered into cultured cells using different transfection agents, like the peptide-mediated delivery reagent Endo-Porter (EPEI) that provides good transfection efficiencies while preventing cell toxicity^49,50^.

In this work we designed and used PMOs to modulate the alternative splicing of E23a of the *NF1* gene in the PC12 neuronal differentiation system. We show that PMOs are useful tools to affect and study alternative splicing of an RNA of interest by forcing the exclusion or inclusion of targeted exons, without the need for endogenous gene editing and while maintaining the physiological levels of gene expression. Furthermore, PMO-mediated targeted splicing changes can be used to study the role of individual isoforms in dynamic processes such as differentiation, as demonstrated here by using *NF1* alternative splicing as a model.

## Results

### Cell-based systems to study the alternative splicing of exon 23a (E23a) of the *NF1* gene

**Figure 1:**
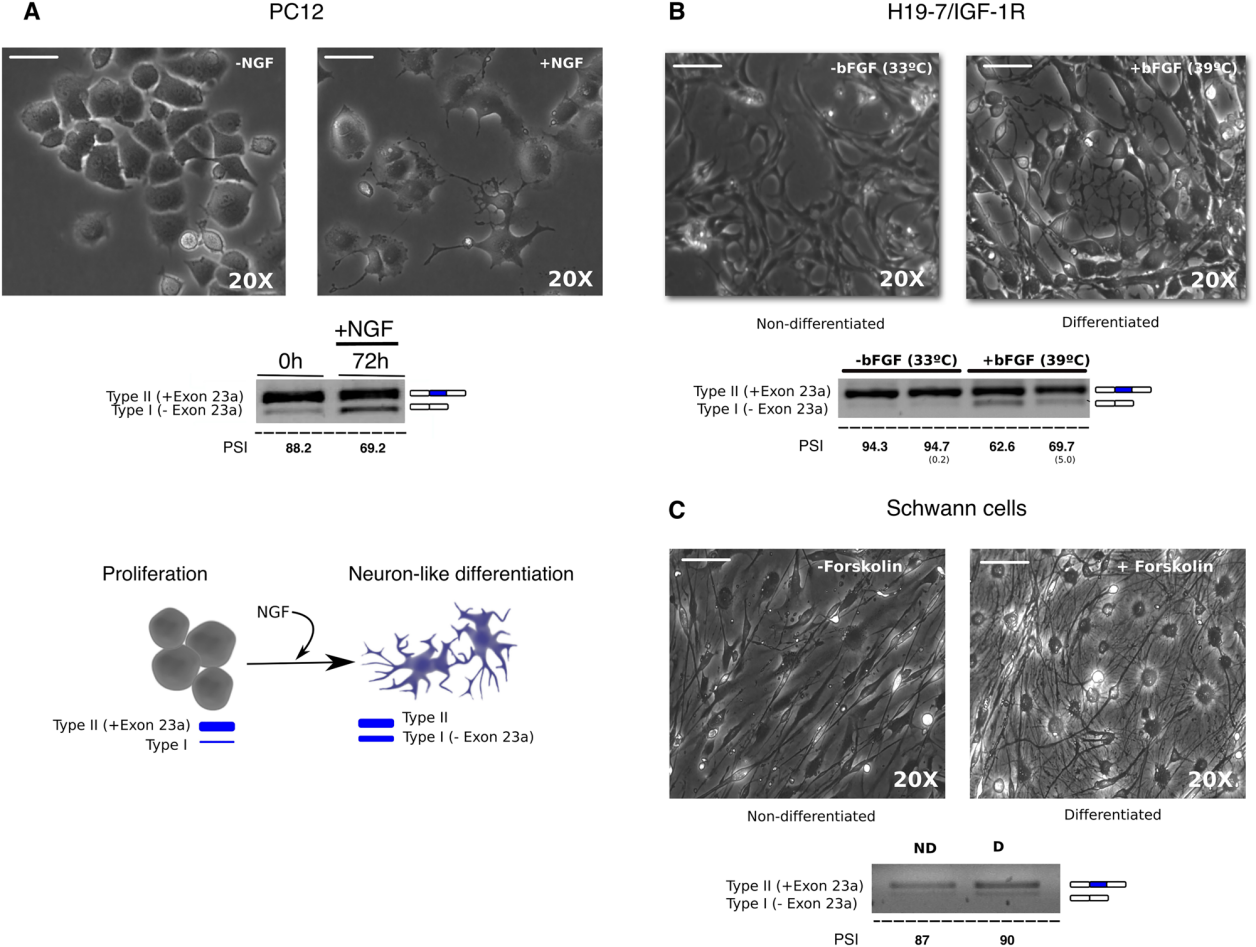
Analysis of the alternative splicing of E23a of the *NF1* gene in different cellular models. (**A**) Top: Phase-contrast images of PC12 cells without (left) or with (right) NGF. Bottom: Schematic representation of E23a alternative splicing during PC12 neuronal differentiation and RT-PCR time course analysis of the switch from *Nf1* type II (+E23a) to type I (-E23a) transcripts after NGF addition to PC12 cells. (**B**) Top: Phase contrast images of H19-7/IGF-IR cells without (left) or with (right) differentiation conditions. Bottom: RT-PCR analysis of *Nf1* type II (+E23a) switch to type I (-E23a) transcript at 24 h is shown. (**C**) Top: Phase contrast images of proliferating nerve-derived primary Schwann cells (left) and differentiating (right) conditions. Bottom: RT-PCR analysis of *NF1* isoform switch is illustrated. For all phase-contrast images scale bar is 30 μm. E23a splicing was quantified as the percent spliced-in (PSI) (standard deviation in parentheses). All supporting uncropped gels can be found in Supplementary Fig. S1.

To design and test the use of Phosphorodiamidate Morpholino Oligomers (PMOs) for modulating the alternative splicing of exon 23a (E23a) of the *NF1* gene, we first evaluated the potential use of different cell-based model systems: two neuronal differentiation systems (pheochromocytoma-derived PC12 cells and embryonic hippocampal H19-7/IGF-IR cells) and nerve-derived Schwann cell (SC) cultures from an NF1 individual. PC12 cells can be stimulated to differentiate into sympathetic-like neurons in the presence of Nerve Growth Factor (NGF)^51^. The use of NGF triggers cell division arrest, neurite extension and increases synthesis of several neurotransmitters^52^. In addition, during differentiation there is a partial switch in *Nf1* isoforms from type II transcripts (E23a included) to the expression of type I transcripts (E23a excluded)^28^. We analyzed the extent and dynamics of E23a alternative splicing during PC12 differentiation in our hands (Fig. 1A). We also analyzed the alternative splicing of E23a during the differentiation of rat embryonic hippocampal H19-7/IGF-IR. Upon the addition of basic Fibroblast Growth Factor (bFGF) and a temperature increase, H19-7/IGF-IR cells extended neurites and changed morphology similarly to PC12 differentiating cells (Fig. 1B). In this cell line, the switch from type II to type I isoform was already observed at 24 h after bFGF-triggered differentiation (Fig. 1B). Finally, we also evaluated E23a alternative splicing in nerve-derived primary Schwann cells (SCs) after a continuous exposure to high concentrations of forskolin, an elevator of cAMP through adenylate cyclase stimulation. We observed a slight increase in type II isoform expression in differentiated SCs (Fig. 1C), in accordance with previous results^4^. With these analyses, we evidenced that the alternative splicing of E23a participates in a different manner in distinct cell differentiation processes. To test the applicability of PMOs on E23a alternative splicing analysis, we selected the PC12 differentiation model, as it was the most amenable cell culture system. However, it should be noted that the PC12 differentiation system is very sensitive to the specific conditions of the cells (cell density, media, manipulation, etc.) and any slight change in these conditions affect the degree of differentiation. We observed different PC12 differentiation outputs, regarding cell morphology and degree of *Nf1* alternative splicing, in independent experiments. However, within each experiment, samples preserved the same relative relationship, and quantification of the degree of E23a splicing facilitated a relative comparison.

### Design and use of PMOs to induce the skipping or inclusion of E23a while maintaining physiological *NF1* levels

**Figure 2:**
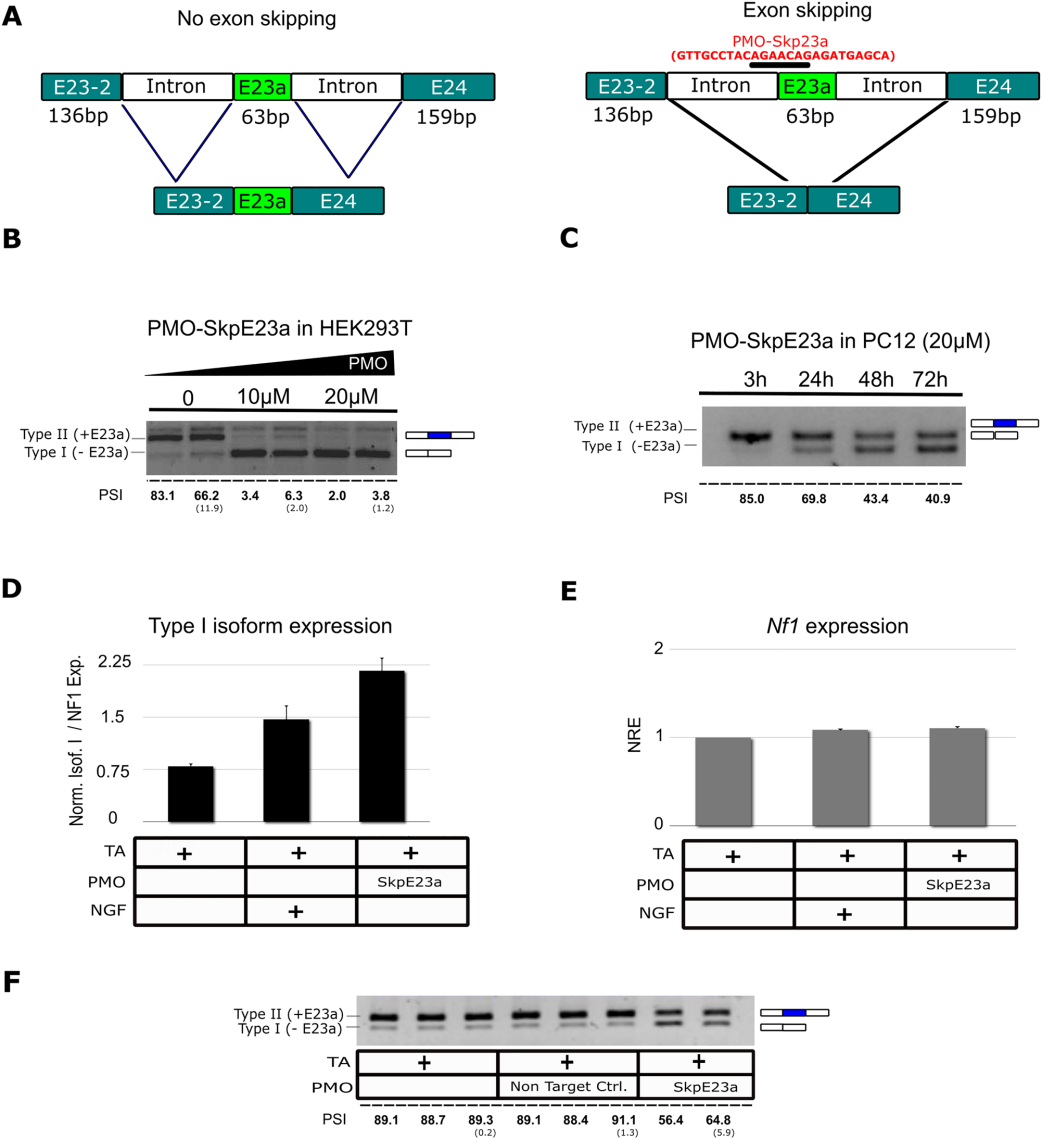
Design of a PMO to force the skipping of E23a and experimental conditions set up. (**A**) Schematic diagram of E23a skipping strategy using PMO-SkpE23a (in red) targeting its acceptor splice site. (**B**) Dose–response analysis at different PMO-SkpE23a concentrations in HEK293T cells measured by RT-PCR. (**C**) Time course experiment using PMO-SkpE23a in PC12 cells measured by RT-PCR. (**D**) Relative mRNA isoform type I (-E23a) expression normalized by the total *Nf1* expression at 72 h, measured by RT-qPCR. (**E**) Total *Nf1* expression levels at 72 h after PMO addition, measured by RT-qPCR. (**F**) Expression of Type II/I isoforms in PC12 cells treated with transfection agent (TA), a non-targeting control PMO and PMO-SkpE23a, 72 h after PMO addition and measured by RT-PCR. For all plots, bars indicate Mean ± SEM. NRE: Normalized Relative Expression. All supporting uncropped gels can be found in Supplementary Fig. S2. E23a splicing was quantified as the percent spliced-in (PSI) (standard deviation in parentheses).

**Figure 3:**
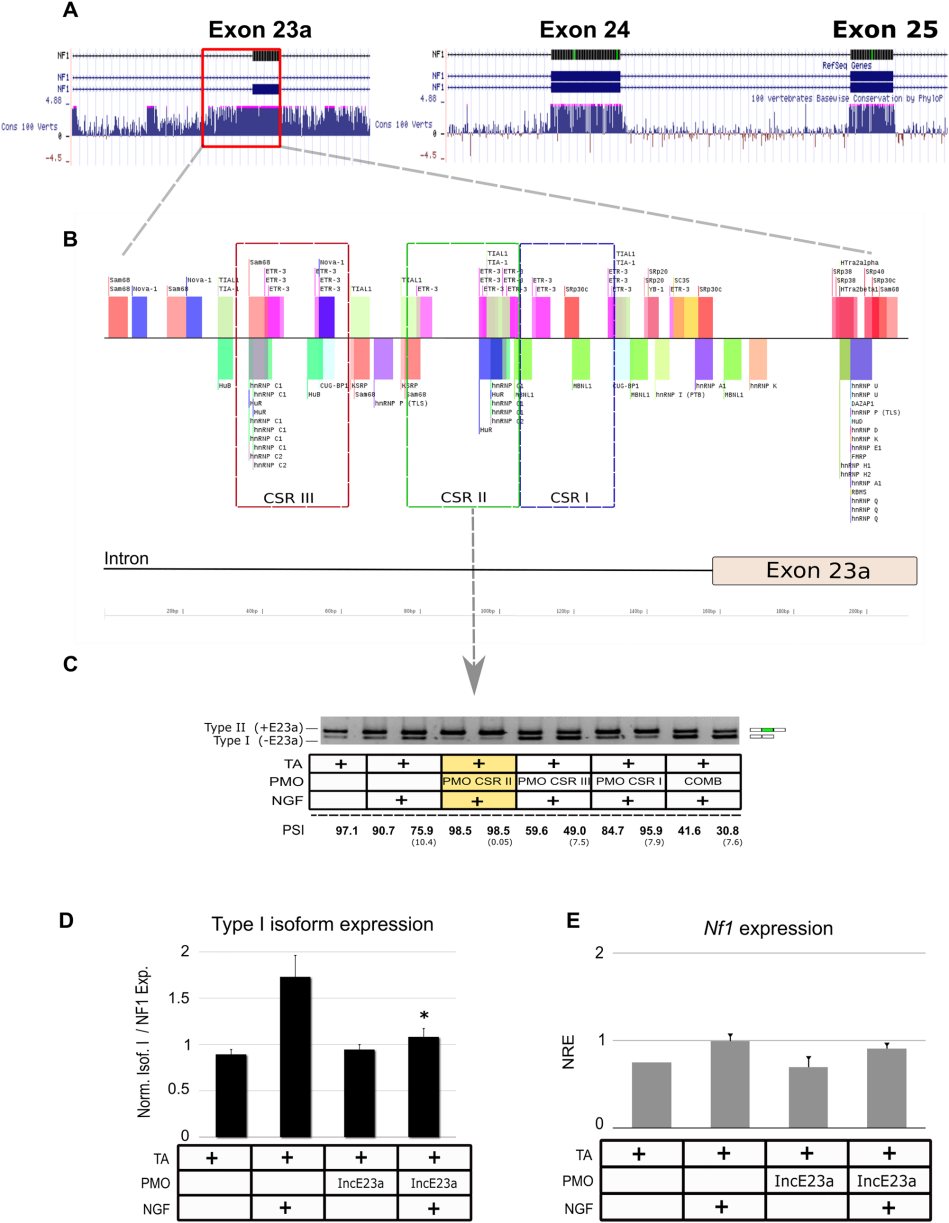
Design of a PMO to force inclusion of E23a and experimental conditions set up. (**A**) Analysis of the degree of sequence conservation of E23a, E24 and E25 of the *NF1* gene and adjacent intronic regions. (**B**) Splicing factors predicted by SpliceAid2 to bind the 5´ intronic region of E23a. Colored boxes represent three Conserved Splicing Regions (CSR) selected to design a PMO to block E23a skipping while preserving a correct splicing. (**C**) Type II and type I *Nf1* isoform expression levels analyzed by RT-PCR using three PMO’s targeting CSR I, II & III and their combination (COMB), 72 h after PMO and NGF treatment. PMO targeting CSR II (highlighted in yellow) was selected as PMO-IncE23a. E23a splicing was quantified as the percent spliced-in (PSI) (standard deviation in parentheses). Supporting uncropped gel can be found in Supplementary Fig. S3. (**D**) Relative isoform type I (-E23a) expression normalized by the total *Nf1* expression by RT-qPCR. (**E**) Total *Nf1* expression levels at 72 h after PMO addition. NRE: Normalized Relative Expression (*Rpl29* and *Rpl19* were used as reference genes). For all plots, bars represent Mean ± SEM. *P < 0.05. TA: transfection agent.

We designed and tested in vitro antisense PMOs to induce the skipping (PMO-SkpE23a) or the inclusion (PMO-IncE23a) of E23a (Figs. 2 and 3). PMO-SkpE23a was designed to specifically bind to the acceptor-splicing site of E23a within the intron–exon junction (Fig. 2A). We first performed a dose–response assay in the highly transfectable HEK293T cell line (Fig. 2B). We tested different concentrations of PMO and successfully induced almost complete E23a skipping at lowest concentration tested of PMO-SkpE23a after 24 h. After adjusting the concentration for PC12 cells, we then tested the efficacy of 20 µM concentration of PMO-SkpE23a to induce E23a skipping in a time course experiment, achieving high levels of E23a skipping already after 24-48 h (Fig. 2C). We also analyzed by RT-qPCR the impact of PMO-SkpE23a on E23a skipping compared to the effect of NGF treatment after 72 h (Fig. 2D) and in the endogenous expression of *Nf1* (Fig. 2E). We finally tested the specificity of the designed PMO-SkpE23a on E23a modulation by comparing its skipping efficacy with that of a non-targeting PMO control (NTC) (Fig. 2F). Results indicated that PMO-SkpE23a treatment was able to induce E23a skipping in a specific manner even at higher levels than NGF alone, while maintaining the endogenous expression levels of *Nf1*.

The PMO-IncE23a was designed to enhance the inclusion of E23a, a more complicated task than forcing the skipping of an exon, due to the high number of splicing factors and the complex regulation of alternatively-spliced exons. We first analyzed the degree of conservation of the intronic sequences surrounding E23a compared to adjacent exons (Fig. 3A). We observed that E23a had a highly conserved intronic region at 5′- and 3′- expanding further away from the coding sequence compared to adjacent exons, as previously described^53^ (Fig. 3A). Non-coding conservation has also been described in the intronic regions adjacent to other important tissue regulated exons^54–56^. Within these conserved intronic regions, we set out to determine the potential binding sites for E23a splicing factors. An in silico analysis identified an enrichment in AU-rich and UG-rich sequences, binding sites for Hu and CELF proteins, E23a skipping promoters^11^, and TIA-1/R or MBNL proteins, E23a inclusion promoters^12,13^ (Fig. 3B). We selected three regions within the 5′ intronic sequence of E23a that concentrated most of the binding factors that promote E23a exclusion (mainly protein families Hu and CELF). These regions were named Conserved Splicing Regions (CSRs). We designed three distinct PMOs to bind to these regions to putatively block the binding of these factors. We then tested their performance in PC12 cells after NGF addition, to assess their capacity to block NGF-triggered E23a skipping. Some of the PMOs induced the skipping rather than the inclusion of E23a, but one of them, targeting CSR II, was able to retain E23a despite NGF treatment of PC12 cells (Fig. 3C). We measured this exon retention quantitatively by RT-qPCR (Fig. 3D), and also confirmed that the use of PMO-IncE23a was not significantly altering the endogenous expression of the *Nf1* gene (Fig. 3E). The different effect on E23a splicing of the three tested PMOs, the specific skipping capacity of PMO-SkpE23a and the lack of E23 splicing effect of an NTC PMO, provided different evidenced of the specificity of the designed E23a PMOs. NTC had no effect on E23a splicing (Fig. 2F; Supplementary Fig. S5A) neither changed the morphology of PC12 cells (Supplementary Fig. S6) or signaling pathways (Supplementary Figs. S7 and S12A,B,C), behaving as the addition of TA only. However, RNA-seq analysis of NTC PMO treated cells, revealed a certain change in gene expression compared to TA alone (data not shown). Given the control on E23a splicing specificity provided by the two antagonistic splicing-regulating E23a PMOs, we decided to use TA alone as control conditions.

### Impact of E23a alternative splicing modulation by PMOs on NGF-mediated PC12 neuronal differentiation

**Figure 4:**
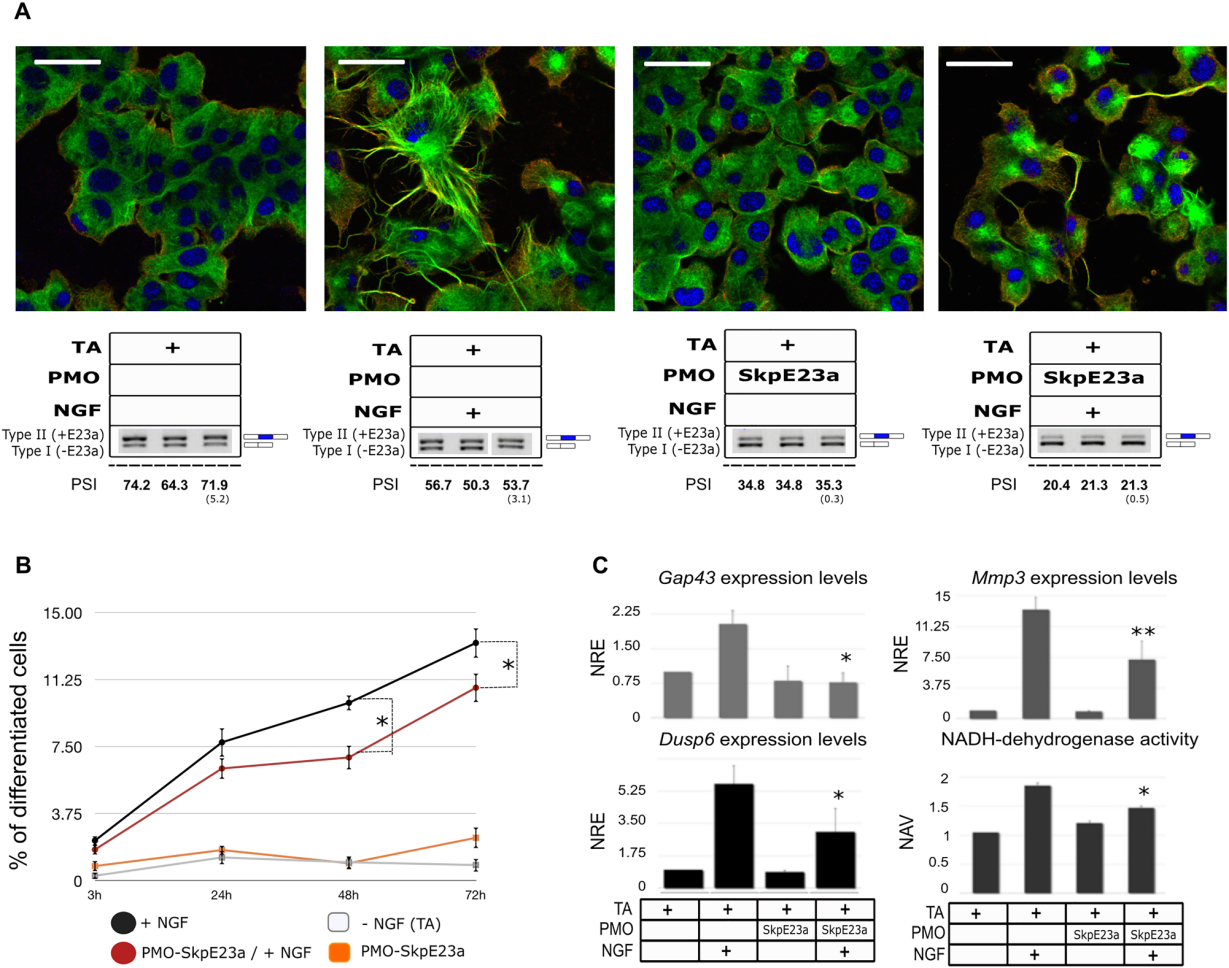
Impact of forcing E23a skipping using PMO-SkpE23a on NGF-induced PC12 neuronal differentiation. (**A**) Top: Representative images of a double immunofluorescent staining of NGFR (red) and α-tubulin (green) of PC12 cells after 72 h at different experimental conditions (scale bar = 40 μm). Bottom: Box specifying the different experimental conditions and the type II/I isoform expression ratio measured by RT-PCR. E23a splicing was quantified as the percent spliced-in (PSI) (standard deviation in parentheses). Supporting whole gel image can be found in Supplementary Fig. S4. (**B**) Time course analysis of the percentage of differentiated cells per field for each experimental condition. Each point represents the Mean ± SEM. *P < 0.05, paired *t*-test versus cells treated only with NGF. (**C**) Expression of the neuronal differentiation markers *Dusp6*, *Gap43*, *Mmp3* analyzed by RT-qPCR and the enzymatic activity of NADH-dehydrogenase at the different experimental conditions set. NRE: Normalized Relative Expression (*Rpl29* and *Rpl19* were used as reference genes). NAV: Normalized Absorbance Values. Bars indicate Mean ± SEM. *P < 0.05, **P < 0.01, paired *t*-test versus cells treated only with NGF. TA: transfection agent.

**Figure 5:**
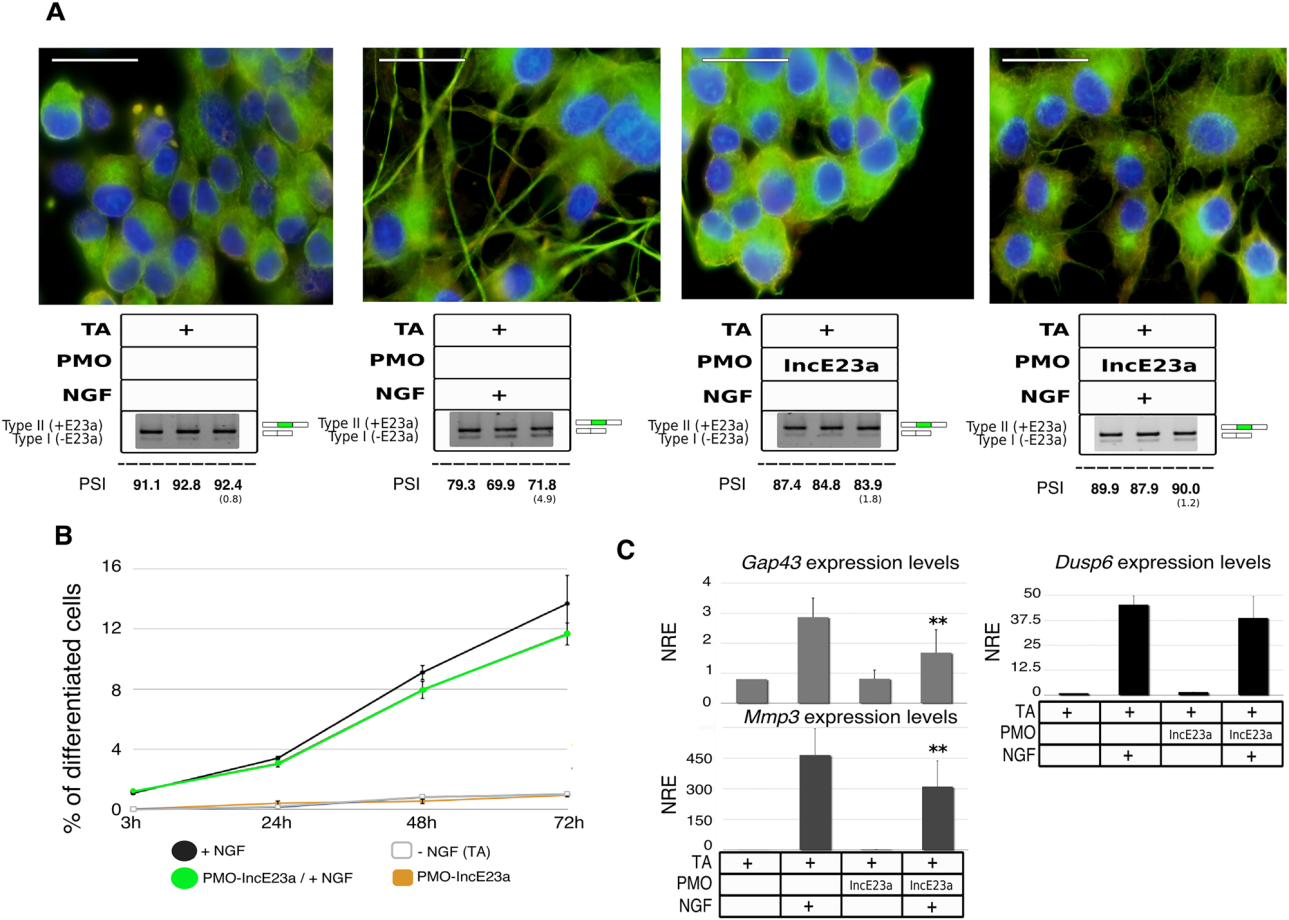
Impact of forcing E23a inclusion using PMO-IncE23a on NGF-induced PC12 neuronal differentiation. (**A**) Top: Representative images of a double immunofluorescent staining of NGFR (red) and α-tubulin (green) of PC12 cells after 72 h at different experimental conditions (scale bar = 30 μm). Bottom: Box specifying the different experimental conditions and the type II/I isoform expression ratio measured by RT-PCR. E23a splicing was quantified as the percent spliced-in (PSI) (standard deviation in parentheses). Supporting whole gel images can be found in Supplementary Fig. S5A. (**B**) Time course analysis of the percentage of differentiated cells per field for each experimental condition. Each point represents the Mean ± SEM. Complementary neurite thickness analysis can be found in Supplementary Fig. S5B. (**C**) Expression of the neuronal differentiation markers *Dusp6*, *Gap43* and *Mmp3* at the different experimental conditions set, analyzed by RT-qPCR. NRE: Normalized Relative Expression (*Rpl29* and *Rpl19* were used as reference genes). Bars indicate Mean ± SEM. **P < 0.01, paired *t*-test against cells treated only with NGF. TA: transfection agent.

After designing and setting up of E23a PMOs, we tested their effect on PC12 neuronal differentiation. As determined by our initial time-course experiment (Fig. 2C), PMO-SkpE23a treatment had an earlier and larger effect on the levels of E23a skipping (i.e. a type II to type I switch) compared to NGF treatment alone in PC12 cells (Fig. 1A). We first evaluated the percentage of PC12 differentiated cells according to neurite generation 72 h after NGF treatment (Fig. 4A,B). Differentiated cells were considered when at least one neurite acquired a length equal or longer than the cell body diameter (see M&M section). PMO-SkpE23a treatment reduced the percentage of differentiated cells and on those differentiated, reduced the formation of multiple and branched neurites (Fig. 4A,B). Furthermore, the induction of E23a alternative splicing in the absence of NGF did not induce neuronal differentiation of PC12 cells. In concordance with the reduced and altered neurite formation, PMO-SkpE23a also reduced the expression of the neuronal markers *Gap43*, *Mmp3* and *Dusp6* and the enzymatic activity of NADH-dehydrogenase (Fig. 4C) on NGF treated cells. We also used PMO-IncE23a to avoid NGF-induced isoform switch from Type II (+E23a) to Type I (-E23a). Again, we evaluated PC12 differentiation 72 h after NGF treatment. The addition of PMO-IncE23a did not change the percentage of cells that generated neurites but clearly reduced neurite thickness (Supplementary Fig. S5B) and their apparent robustness compared to NGF treatment alone (Fig. 5A,B). Consistent with an altered differentiation, expression of neuronal markers was also altered (Fig. 5C) although not to the same extent as using PMO-SkpE23a.

**Figure 6:**
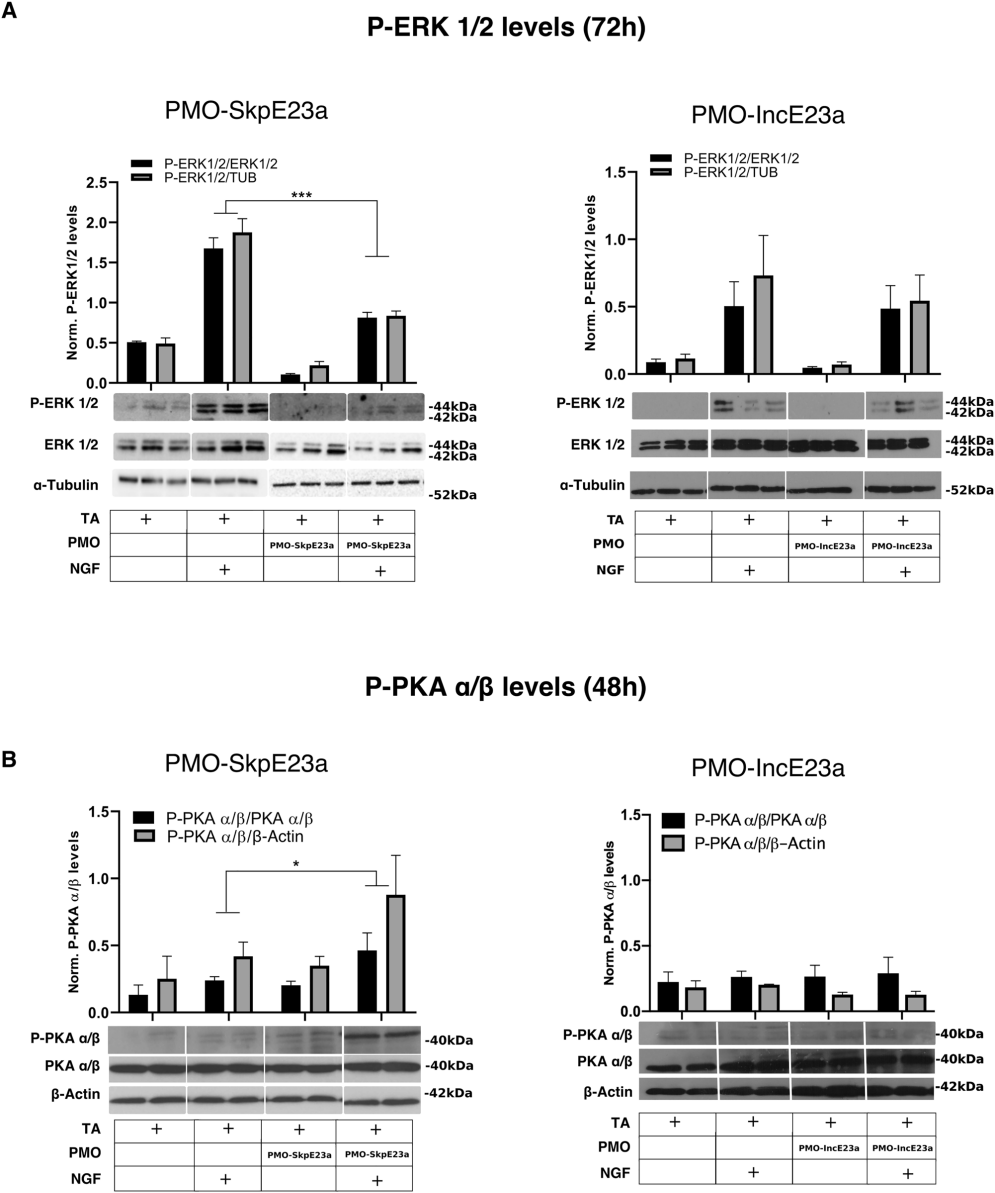
Effect of E23a skipping or inclusion by PMO treatment on the activation of ERK and PKA during NGF-induced PC12 neuronal differentiation. (**A**) Western blot analysis and quantification of P-ERK1/2, total ERK1/2 and alpha-tubulin levels after forcing E23a skipping (PMO-SkpE23a) or inclusion (PMO-IncE23a) at 72 h after NGF treatment at different experimental conditions summarized in a box below each graph/western. ***P < 0.001, paired *t*-test versus cells treated only with NGF. Bars indicate Mean ± SEM. (**B**) Western blot analysis and quantification of P-PKAαβ, total PKAαβ and β-actin, at the same conditions as in (**A**), but 48 h after NGF treatment. *P < 0.05, paired *t*-test versus cells treated only with NGF. Bars indicate Mean ± SEM. TA: transfection agent. Supporting whole gel images can be found in Supplementary Fig. S7.

To further explore the molecular consequences of *NF1* alternative splicing modification by PMOs on PC12 neuronal differentiation, we analyzed the status of the MAPK/ERK and cAMP/PKA signaling pathways. Consistent with a higher RAS-GAP activity of Type I (-E23a) isoform, we identified reduced levels of ERK 1/2 phosphorylation in PC12 cells treated with PMO-SkpE23a and with or without NGF, compared to their respective controls, 72 h post NGF addition (Fig. 6A). We did not observe significant changes in the levels of ERK phosphorylation in PMO-IncE23a treated cells compared to controls (Fig. 6A). We also measured the activity of the cAMP/PKA signaling pathway by western blot of phosphorylated PKA α/β (P-PKA α/β). P-PKA α/β levels slightly increased in PC12 cells upon NGF treatment. In the presence of PMO-SkpE23a, the levels of P-PKA α/β were higher upon NGF treatment than in NGF-treated PC12 cells alone (Fig. 6B). PMO-IncE23a treatment had no effect on PKA activation on PC12 cells (Fig. 6B).

Taken together, these results indicated that any alteration of the natural *NF1* isoform switch produced by NGF in PC12 cells altered their neuronal differentiation phenotype morphologically, at the level of neuronal marker expression and on the regulation of *NF1*-related signaling pathways.

### Dynamic modification of E23a alternative splicing by PMOs in PC12 neuronal differentiation

One of the advantages of using PMOs to study the impact of alternative splicing on cell physiology is their flexibility of use: different concentrations of PMO with different quantitative effects on splicing can be used; its treatment can be reversed; its addition can be performed at different time points using the same cells. This flexibility facilitates the study of physiological processes in a dynamic way. To test this, we designed an experiment in which we analyzed the effect of inducing the alternative splicing of E23a before adding the biological stimulus (NGF) responsible for the natural *NF1* alternative splicing during PC12 cells differentiation (Supplementary Fig. S8). By inducing type I isoform (-E23a) before NGF treatment, we were conferring the maximum Ras-GAP activity to *NF1* expression before the induction of PC12 differentiation. To have a more complete experimental framework, we also designed a new set of PMOs (PMO-SkpE14; Supplementary Fig. S9), to force the skipping of an out-of-frame exon, exon 14 (E14). By doing this, a premature stop codon was formed, inducing the nonsense mediated decay (NMD) machinery, highly reducing neurofibromin expression (Supplementary Fig. S9D) and thus, expecting a reduction in the overall Ras-GAP activity.

**Figure 7:**
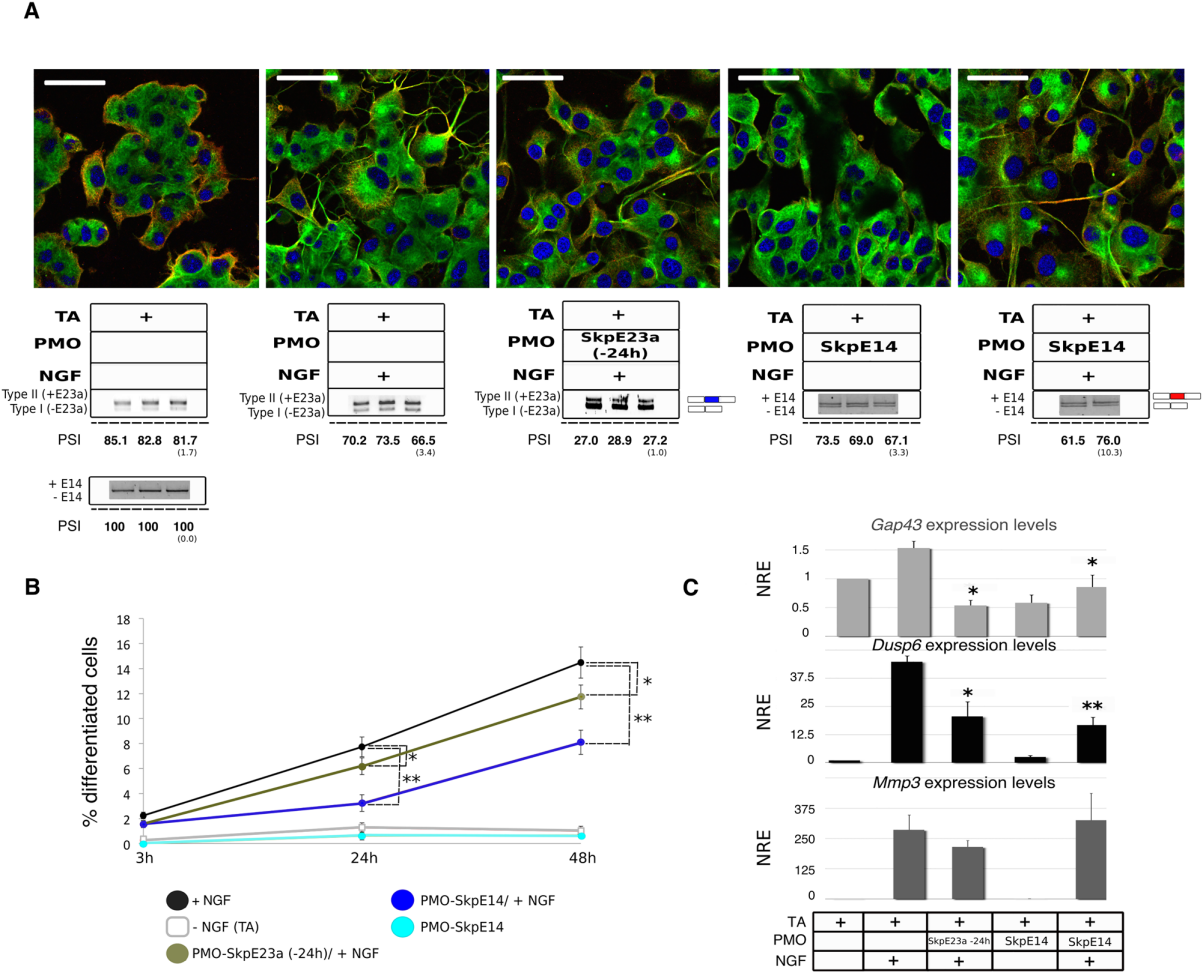
Impact of inducing *NF1* isoform switch before NGF-triggered differentiation of PC12 cells. (**A**) Top: Representative images of a double immunofluorescence staining of NGFR (red) and α-tubulin (green) of PC12 cells after 48 h at different experimental conditions (scale bar = 62 μm). Bottom: Box specifying the different experimental conditions and either the type II/I isoform expression ratio or the levels of E14 skipping measured by RT-PCR. For detection of E14 skipping, cells were treated with puromycin. E23a and E14 splicing was quantified as the percent spliced-in (PSI) (standard deviation in parentheses). Supporting whole gel images can be found in Supplementary Fig. S10. (**B**) Time course analysis of the percentage of differentiated cells per field for each experimental condition. Each point represents the Mean ± SEM. *P < 0.05, **P < 0.01, paired *t*-test versus cells treated only with NGF. (**C**) Expression of neuronal differentiation markers *Dusp6*, *Gap43*, *Mmp3* at different experimental conditions, analyzed by RT-qPCR. NRE: Normalized Relative Expression (*Rpl29* and *Rpl19* were used as reference genes). Bars indicate Mean ± SEM. *P < 0.05, **P < 0.01, paired *t*-test versus cells treated only with NGF. TA: Transfection agent.

PC12 cells were treated with PMO-SkpE23a and with PMO-SkpE14 24 h before NGF treatment and we analyzed neuronal differentiation capacity in a time course at morphological, gene expression and signaling levels. Forcing the switch from type II to type I *NF1* isoform before NGF treatment reduced the percentage of differentiated cells and its morphological complexity, with neurites being straighter, thicker and less branched (Fig. 7A,B) similarly to adding PMO-SkpE23a at the same time as NGF (Fig. 4A,B). In addition, the expression of neuronal markers was also reduced (Fig. 7C). A similar but enhanced alteration on PC12 differentiation was obtained when cells were treated with NGF and PMO-SkpE14, with a decreased percentage of differentiated cells, with a reduced number of neurites, being less complex and longer (Fig. 7A,B; Supplementary Fig. S11A). In fact, differentiated cells showed an increase in varicosite thickness along the neurite extensions (Supplementary Fig. S11B). The expression of neuronal differentiation markers was also altered (Fig. 7C). However, PMO-SkpE14 elicited a significant increase in early apoptotic cells that was partially reverted by the NGF-induced cell cycle arrest of PC12 cells (Supplementary Fig. S11C). Thus, results regarding the use of PMO-SkpE14 have to be circumscribed to the cells that did not undergo apoptosis, being biased in this regard.

**Figure 8:**
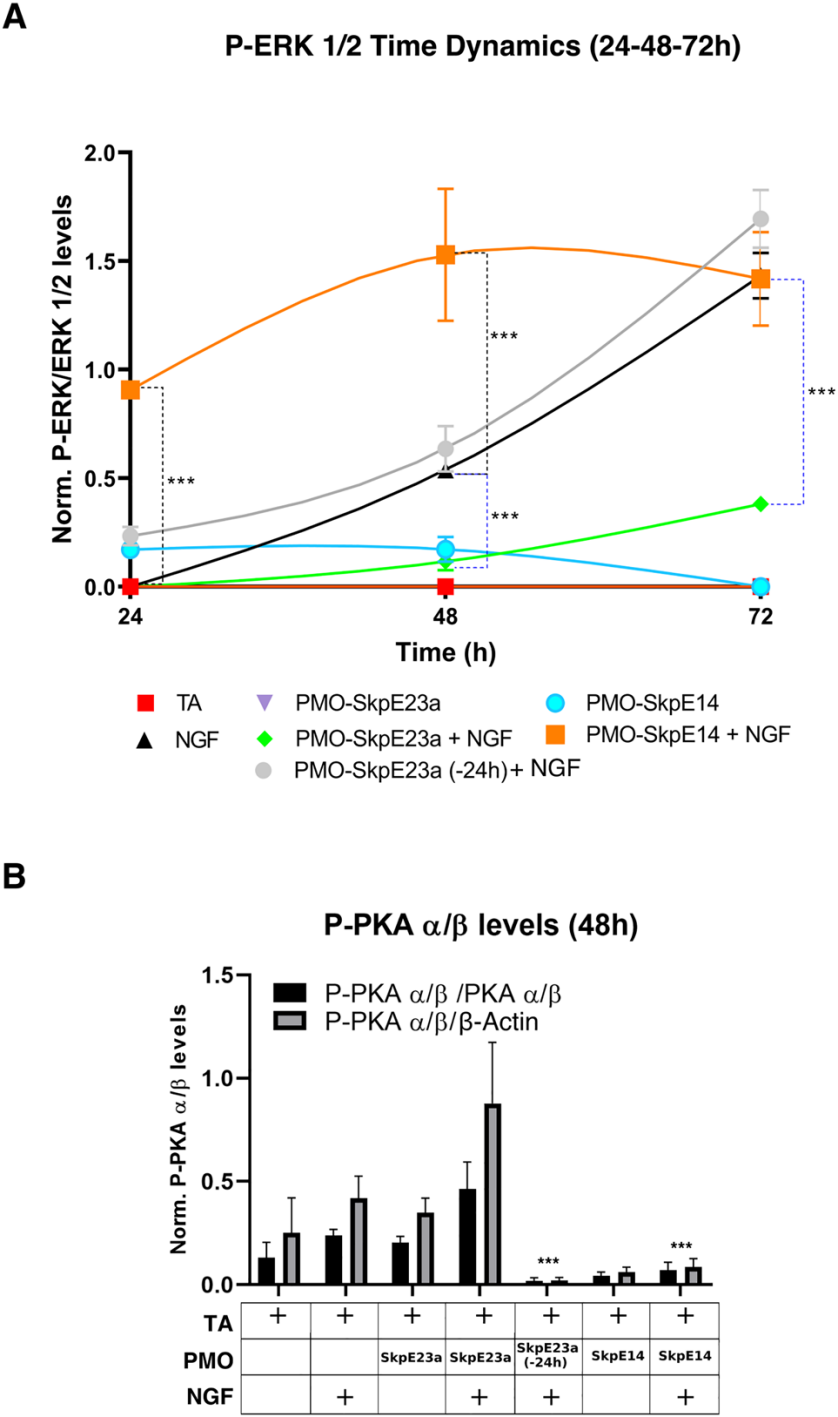
Effect on ERK and PKA activation of inducing *NF1* isoform switch before NGF-triggered differentiation of PC12 cells. (**A**) Time course expression analysis by Western blot of P-ERK1/2 levels normalized to total ERK1/2 levels using different PMOs and PMO conditions. Differences between PMO-SkpE23a added 24 h before NGF treatment (-24 h), PMO-SkpE23a and PMO-SkpE14 added at the same time as NGF treatment were evaluated by paired *t*-test versus cells treated only with NGF. Each point represents the Mean ± SEM. ***P < 0.001. (**B**) Expression analysis by Western blot and quantification of P-PKA and total PKA using different PMOs and PMO conditions. Differences were evaluated by paired *t*-test versus cells treated only with NGF. Bars indicate Mean ± SEM. ***P < 0.001. Supporting whole gel images can be found in Supplementary Fig. S12.

In addition, we analyzed the status of the MAPK/ERK and cAMP/PKA signaling pathways in these experimental conditions (Fig. 8 and Supplementary Fig. S12), adding also a condition in which PMO-SkpE23a was included at the same time as NGF, for comparison purposes. As before (Fig. 6A) P-ERK 1/2 levels were significantly reduced when PMO-SkpE23a was added at the same time as NGF along the time-course experiment, according to an increased Ras-GAP activity. In clear contrast and surprisingly, in conditions where PMO-SkpE23a was added 24 h before NGF, ERK phosphorylation levels were similar to control cells treated with NGF, reaching levels at 72 h similar to those in PC12 cells treated with PMO-SkpE14, with low levels of neurofibromin and thus low Ras-GAP activity (Fig. 8A). Furthermore, we also measured PKA phosphorylation levels at 48 h after NGF treatment. When PMO-SkpE23a was added at the same time as NGF there was a clear increase in PKA phosphorylation (Fig. 8B and Supplementary Fig. S12D) as observed previously (Fig. 6B). Again, this result contrasted with conditions where PMO-SkpE23a was added 24 h before NGF, in which PKA was activated below control levels, similarly to the effect triggered by PMO-SkpE14, in conditions with depleted neurofibromin (Fig. 8B). These results in one hand supported the view that there was an inverse relationship between the function of neurofibromin and the regulation of either the MAPK/ERK pathway or the cAMP/PKA pathway during PC12 neuronal differentiation. On the other hand, they suggested an apparent desensitization of neurofibromin function if the isoform switch towards a higher GAP activity (E23a-less type I isoform) was produced before the addition of NGF. Consistent with this view, RNA-seq analysis of these conditions (Supplementary Fig. S13) revealed a transient transcriptional activation of genes due to PMO-SkpE23a addition alone (Supplementary Fig. S13A). Many of these genes were NGF-response genes (Supplementary Fig. S13B). This differential global expression status at the time of NGF addition may explain the differences in signaling and physiological readouts in PC12 cells under these two different conditions, and may show a pathway feedback triggered by NGF and dependent on neurofibromin function. At the same time, these results highlight the importance of the timing at which genetic perturbations are made in this PC12 neuronal differentiation model system, since although being the exact same system, results and their interpretation can be very different.

## Discussion

The present work illustrates the utility of PMOs to study alternative splicing. PMOs allow the preservation of physiological gene expression conditions and can be used in a highly flexible way, facilitating the study of dynamic processes and partially overcoming static genetic methods. In addition, results obtained by applying PMOs to the study of the alternative splicing of *NF1* E23a highlight the importance of a precise quantitatively and time-dependent regulation of E23a splicing along the neuronal differentiation process. Finally, it constitutes an interesting example of how the alternative splicing of a single gene can influence cell fate by the fine, coordinated, and time-dependent regulation of different signaling pathways.

Antisense technology has been scarcely used for exploring the biological processes related to the natural alternative splicing of genes^45–47^. In this work, all designed PMOs had an incomplete but reasonable efficiency regarding the degree of induced exon skipping or inclusion already 24 h after PMO treatment. The use of PMOs to force exon inclusion has been explored to a lesser extent than for the skipping of an exon^57–59^ and even lesser in the context of alternative splicing. The analysis of intronic sequence conservation^53^, as well as previous information on both *cis*-acting sequences and *trans*-acting factors (Hu and CELF; and TIA-1/R and MBNL proteins)^11–13,60^ were of especial importance for the identification of a PMO that forced the inclusion of E23a in conditions of NGF-triggered E23a skipping. Moreover, an advantage of using E23a PMOs was the successful modulation of E23a splicing without significantly altering the physiological levels of *Nf1* mRNA expression, in contrast to the use, for instance, of over expression plasmids. Another advantage of PMOs is its flexible use regarding the time of exposure and its duration, its reversibility and the concentration used in relation to the degree of splicing modulation. This flexibility allowed us to study the impact of E23a alternative splicing in PC12 neuronal differentiation overcoming static genetic methods, such the generation of genetically modified cell lines^29^ and being able to observe more subtle and complex regulations, such as the feed-back regulation of signaling pathways (see below). The developed PMOs can be applied to other NF1 in vitro and in vivo models to better understand the role of alternative E23a splicing while preserving physiological conditions. Furthermore, the same type of approach can be applied to other alternatively spliced genes in the context of other biological conditions.

PC12 neuronal differentiation is a complex physiological process that implicates the activation of a network of signal transduction pathways triggered by NGF. These pathways integrate into a transcriptional response that regulate both signaling and morphological and physiological changes^32–34,51,61^. We tested the utility of designed PMOs to study the implication of *NF1* E23a alternative splicing in neuronal differentiation. We first evidenced that mimicking the switch from type II to type I neurofibromin by PMO-SkpE23a alone, without NGF addition, did not induce PC12 cells to undergo neuronal differentiation. We also observed that the use of each *NF1* E23a PMO (PMO-SkpE23a and PMO-IncE23a) elicited a different perturbation of the differentiation process, although in both cases implicated the formation of neurites (either in number, complexity or robustness), the alteration of the expression of neuronal differentiation markers, and the deregulation of both MAPK/ERK and cAMP/PKA pathways. Despite the different elicited responses, the use of *NF1* E23a PMOs clarified that any quantitative or time-dependent alteration of the NGF-triggered natural switch from type II to type I neurofibromin, impacted on the correct neuronal differentiation of PC12 cells at signaling, gene expression and morphological levels.

Regulatory networks driving complex and dynamic physiological responses, such as neuronal differentiation, are difficult to study. The role of *NF1* E23a alternative splicing on neuronal differentiation has been studied using the overexpression of specific isoforms or generating mouse cell lines with both *Nf1* alleles with or without E23a^29^. However, non-physiological or static perturbations may not be the best tools for a fine analysis. Although not perfect, PMOs permit a greater flexibility in system perturbation. For instance, PMOs allowed us to force the skipping of E23a towards type I neurofibromin, with the highest Ras-GAP activity, before the addition of NGF to PC12 cells. In these initial conditions, after NGF addition we expected a reduced level of ERK1/2 activation, at least similar to conditions in which NGF and PMO-SkpE23a were added simultaneously. Surprisingly, the levels of ERK1/2 phosphorylation were similar to NGF-treated control cells and comparable to those exhibited by PC12 cells in which neurofibromin was knockdown by PMO-SkpE14 (Fig. 8). Similarly, when PMO-SkpE23a was added 24 h before NGF, PKAα/β activation was reduced, as in PMO-SkpE14-treated cells, and not activated as in conditions where NGF and PMO-SkpE23a were added simultaneously. All these results seemed to point to an apparent desensitization of neurofibromin function in PC12 cells if exposed to neurofibromin with a high Ras-GAP capacity before NGF addition and to a hypothetical feed-back loop to sense pathway activation. Consistent with this hypothesis, in PC12 cells exposed for 24 h to PMO-SkpE23a, we identified an upregulation of NGF response genes. Thus, these cells had a different NGF-related transcriptional profile at the moment NGF was added to the media. Further experiments are needed to confirm the existence of this putative feed-back loop, but PMO flexibility allowed to alert about its possible existence.

MAPK/ERK sustained activation^62^ and its crosstalk with cAMP-dependent signaling pathways^33,34,61^ are key events in PC12 neuronal differentiation. Neurofibromin participates of both pathways^63^ and the time-course dynamics of type II to type I isoform switch in PC12 differentiation correlates with the level of RAS activation^15^. Nevertheless, the whole picture is still incomplete. In the present work we performed a limited analysis of pathway activation (e.g.: no direct measurement of cAMP or Ras-GTP levels). However, despite its limitations, results obtained with the use of E23a PMOs suggest some new insights into the regulation of signaling pathways by neurofibromin. In particular, they suggest a coordinated and opposite regulation of the MAPK/ERK and cAMP/PKA pathway. The addition of PMO-SkpE23a together with NGF resulted in a reduced activation of MAPK/ERK pathway, consistent with an increased neurofibromin GAP activity. But this co-treatment also induced an upregulation of the cAMP/PKA signaling. On the contrary, forcing type II isoform (+E23a) with less neurofibromin GAP activity, resulted in a similar ERK activation and a clear downregulation of the cAMP/PKA pathway. These results clearly illustrated a coordinated an opposite regulation of both pathways. Further analysis is required to understand how both neurofibromin isoforms differentially regulate cAMP/PKA signaling. All together our results suggest that for a correct NGF-triggered PC12 neuronal differentiation, the time-dependent fine regulation of the *NF1* E23a alternative splicing is important. Neurofibromin activity resulting from the *NF1* E23a alternative splicing regulates both MAPK/ERK and cAMP/PKA signaling pathways in an opposite manner, decreasing MAPK/ERK and increasing cAMP/PKA activity, coordinating both signals in a time-dependent way along the differentiation process.

Finally, cAMP/PKA signaling has been shown to be essential for mediating *Drosophila* learning and memory^31^. At the same time, genetically engineered mouse lacking^35^ or retaining E23a^36^ recapitulate *NF1* learning and cognitive disabilities, similar to those of *NF1*(+/−) mice^64–66^, and also similar to the ones observed in NF1 patients. The designed PMOs could be used in an in vivo format, either by a direct delivery or in combination with agents that facilitate to cross the blood–brain barrier^67,68^, to test their impact on learning and memory of WT and *NF1*(+/−) mice.

The present work shows the potential of using PMOs to study alternative splicing, since they preserve physiological gene expression conditions and can be used in a quite flexible way in contrast to static genetic methods. When applied to the study of the role of *NF1* E23a alterative splicing in PC12 neuronal differentiation, PMOs allowed to clarify the precise quantitatively and time-dependent regulation of E23a splicing along the neuronal differentiation, suggested a potential feed-back loop regulation in the NGF-triggered neurofibromin-dependent signaling, and showed the coordinated and opposite regulation of the MAPK/ERK and cAMP/PKA signaling pathways by neurofibromin.

## Materials and methods

### Cell culture

PC12 cells (ATCC CRL-1721) were grown on Collagen IV-coated dishes (25 μg/ml) and maintained in RPMI 1640 medium, supplemented with 10% fetal bovine serum (complete RPMI medium) at 37ºC in 5% of CO_2_. For the NGF treatment, PC12 were plated on Collagen IV-coated dishes in complete medium for 24 h and then switched to RPMI with 1% fetal bovine serum and 50 ng/ml NGF (Promega). H19-7/ IGF-IR (ATCC CRL-2526) were maintained in DMEM supplemented with 10% fetal bovine serum, 200 mg/ml of G418 (Calbiochem) and 1 mg/ml of puromycin (Sigma-Aldrich) at 33ºC in 5% CO_2_. For differentiation experiments, H19-7/IGF-IR cells were grown to confluence and then cells were washed extensively and shifted to the non-permissive temperature of 39ºC with 5% of CO_2_ in DMEM medium supplemented with 1X N2 (Calbiochem) and 10 ng/mL bFGF (Invitrogen). Primary Schwann cells previously isolated from an NF1 patient nerve biopsy, were thawed and cultured on Poly-L-Lysine (0.1 mg/mL) and laminin (4 µg/mL) coated plates in Schwann Cell Media (SCM) + forskolin, and incubated at 37 °C and 10% CO_2_ for 24 h as described previously^69^. Then medium was replaced by SCM without forskolin for 2 days. For proliferative conditions, cells were cultured in SCM + forskolin for 1 day and replaced with SCM for 2–3 days, repeating this process in cycles. For differentiation conditions, cells were cultured with SCM + forskolin constantly for 5 days.

### RNA extraction, quantification and reverse transcription (RT)

Total RNA was extracted using Tripure Isolation Reagent (Roche). Quality and quantity were assessed using NanoDrop spectrophotometer and gel electrophoresis analysis. For an accurate quantification, size and integrity of the RNA was determined with Bioanalyzer (RNA Nano Assay: 25–500 ng/µl), according to manufacturer instructions. Reverse transcription of 1-3 μg of RNA was performed with SuperScript II First-Strand Synthesis System (ThermoFisher Scientific) with random hexamers (Life Technologies, SA) following manufacturer’s instructions.

### E23a alternative splicing analysis and *Nf1* expression levels

For the quantification of E23a alternative splicing (Figs. 2D and 3D) and *Nf1* expression levels (Figs. 2E and 3E) RT-qPCR analysis was performed. Primers used are summarized in Supplementary Table S2. In particular, primers were designed to specifically detect exon 23a skipping (exon 23–2/24 boundary) and inclusion (exon 23–2/23a boundary). In addition, a pair of primers was used to detect the expression of constitutive exon 3, to assess global *Nf1* expression. Expression levels were normalized using *Rpl29* and *Rpl19* reference genes^70^. RT-qPCR was performed using SYBR Green I master mix (Thermo Fisher Scientific) according to manufacturer’s instructions. Reactions were run on Light Cycler 480 II Real Time PCR system (Roche). Conditions for amplification were as follows: 95 °C for 10 min; 40 cycles of 95 °C for 10 s, 59 °C for 30 s, and 72 °C for 20 s; and finally, a melting curve of 95 °C for 5 s and 65ºC for 1 min.

Monitorization of E23a alternative splicing status in all other experiments was performed by RT-PCR using 1 μl of cDNA and running PCR products on a 2% agarose gel. Primers (forward E23-2 and reverse E25, Supplementary Table S2) and PCR conditions were based in the work of Metheny L et al. (1996)^28^ in a 25 cycles reaction. Percent spliced-in (PSI) was calculated as the proportion of type II isoform versus total expression, using band intensities and according to this formula: (PSI) = Type II Isoform/(Type II + Type I isoforms).

### PMO design and treatment conditions

The 25-mer PMOs were designed, synthesized and purified by GeneTools (Philomath, OR, USA) (Supplementary Table S1). PMOs designed to force the skipping of exons 14 (E14) and 23a (E23a) did not require any additional analysis. However, in order to identify the proper target sequences for the design of PMOs forcing the inclusion of E23a, a previous bioinformatic analysis was performed. A sequence comprising E23a and 160 bp of the intron sequence preceding it (hg38, chr17: 31252778–31253000) containing a high degree of homology across species was analyzed using the online tool SpliceAid 2^71^ to determine RNA sequences predicted to be bound by splicing proteins in humans. The SpliceAid 2 database contains human splicing factors expression data and RNA target motifs information. The high level of conservation of intronic and exonic sequences across the E23a region opened the possibility that the molecular mechanisms and splicing regulation were conserved between human and rat (PC12 cells). Considering the predicted binding sites and previously published data^11,12^, we identified intronic sequences of 20–30 bp with potential regulatory properties for its inclusion and selected three of these regions, the conserved splicing regions (CSR) I, II and III, for the design of 25-mer complementary PMOs to sterically block the potential binding of the regulatory proteins. These PMOs were functionally tested in vitro.

Endo-Porter (GeneTools) was used to deliver PMOs into cells, according to the manufacturer’s instructions. For the PMO treatment cells were seeded at 50% of confluence. The next day, culture medium was replaced with fresh medium containing the indicated concentrations of PMOs. Immediately after, Endo-Porter (6 mM) was added and mixed. For the detection of exon 14 skipping, PC12 cells were treated with 200 μg/ml puromycin (Sigma) 4 h before RNA extraction to inhibit the nonsense-mediated decay (NMD) mechanism. Three biological replicates were done for each condition to be studied.

### Differentiation assays and quantitative morphology

Phase contrast images of living PC12 cells were acquired with a LEICA DMI 6000B inverted microscope. At least 10 independent fields per condition were acquired. Cells with at least one neurite with length equal or longer to the cell body diameter were considered differentiated. We calculated the number of differentiated and undifferentiated cells in each field to obtain the percentage of differentiated cells over total cells in the field. Neurite extension was measured by determining the length of at least 10–20 neurites per field using the ImageJ^72^ (versions 1.45 s and 1.8.0_172; https://imagej.nih.gov/ij/download.html) plugin NeuronJ^73^. Neurite thickness was analyzed using ImageJ^72^ (versions 1.45 s and 1.8.0_172; https://imagej.nih.gov/ij/download.html) and measured at three different neurite locations from the cell body (distal, central and apical). The NADH-dehydrogenase activity assay was measured with a Cell Counting Kit-8 (Dojindo), following the manufacturer’s instructions and measuring absorbance at 450 nm using a microplate reader (Spectra Max 34OPC, Molecular Devices). The absorbance values were normalized with the total number of cells per condition.

Expression levels of neuronal markers *Gap43*, *Mmp3* and *Dusp6* were analyzed by RT-qPCR using the same conditions as described above for *Nf1*, also normalized using *Rpl29* and *Rpl19* reference genes^70^.

### Immunofluorescence

Cells were grown on plastic chamber slides, fixed in 4% Paraformaldehyde, permeabilized with 0.1% Triton X-100 for 10 min and blocked for 15 min in 10% of Fetal Bovine Serum (FBS) in PBS. Cells were incubated with α-tubulin (Invitrogen, 1:1000) and NGFR (Advance Targeting systems, 1:100) antibodies in 1% FBS in PBS and incubated 2 h at room temperature (RT). Secondary antibody Alexa Fluor 568 goat anti-rabbit IgG (H + L) (Invitrogen) was used at 1:1000 in PBS (10% FBS) and incubated for 45 min at RT. Glass cover slips were mounted with VECTASHIELD mounting medium with DAPI H-1200 (Vector Laboratories). Images were taken with the confocal microscope Zeiss AxioObserver Z1.

### Cell death analysis

The analysis of apoptotic cells was performed using Annexin-V-Alexa 568 antibody (Roche, Life technologies). In order to differentiate necrotic from positive apoptotic cells, Annexin-V staining was performed in combination with a DNA staining (bisbenzimide Hoechst 33258). The stained cells were analyzed by flow cytometry using BD LSRFortessa cell analyzer.

### Western blot

Cells were lysed in RIPA buffer containing 150 mM NaCl, 1.0% IGEPAL CA-630, 0.5% sodium deoxycholate, 0.1% SDS, 50 mM Tris, pH 8.0. Total protein was determined using the commercially available kit, Pierce BCA Protein Assay Kit (Thermo Scientific), following manufacturer’s instructions. For western blotting, the protein samples (20–30 μg) were separated by size on SDS–polyacrylamide gel, transferred into a PVDF membrane by electroblotting, blocked with 5% non-fat dry milk and subjected to immunoblotting with primary antibodies overnight at 4ºC. All antibodies used were diluted in 3% non-fat dry milk and include P-ERK 1/2 (Cell Signalling diluted 1:2000), ERK 1/2 (Millipore diluted 1:1000), P-PKA α/β (Invitrogen diluted 1ug/ml), PKA α/β (Termo Fisher Scientific diluted 1:500) NF1 c-terminal (Bethyl diluted 1:1000), NF1 n-terminal (Santa Cruz diluted 1:500) and α-Tubulin (Sigma Aldrich 1:1000). Blot was exposed to Horse Radish Peroxidase-conjugated secondary antibody (1 h) and detected with Pierce ECL western Blotting Substrate. Band density was quantified using ImageJ^72^ (versions 1.45 s and 1.8.0_172; https://imagej.nih.gov/ij/download.html).

### Statistical analysis

All functional assays to assess the effect of PMOs were repeated at least three times. We calculated the mean ± SEM for each biological readout and applied Student’s t-test assuming equal variances. An event was considered significant with a *P* value < 0.05. The statistical analysis was performed using PASW Statistics (SPSS Inc. Released 2009. PASW Statistics for Windows, Version 18.0. Chicago: SPSS Inc.; http://www.spss.com.hk/statistics/), while graphical representations were prepared using Prism (version 8.2.1 for MacOS X, GraphPad Software, San Diego, California USA; https://www.graphpad.com/scientific-software/prism/).

### RNA-seq

Total RNA extraction was performed using Maxwell 16 LEV simplyRNA Purification Kit (Promega) according to manufacturer’s instructions. Libraries were prepared starting from 200 ng of RNA per sample with the TruSeq stranded mRNA Illumina procedure. Libraries were quantified using the KAPA library quantitation kit for Illumina GA and sequenced on the Illumina HiSeq 2000 platform with 2 × 100 reads, 4 samples per lane. Sequencing data was quality trimmed with Trimmomatic v0.3 then processed with TopHat v 2.0.9, mapping with Bowtie2 against the *Rattus norvergicus* reference genome Rnor 6.0. The reads overlapping every known gene were counted and the counts matrix was processed in R. Every sample was normalized by the median of counts. A list of differentially expressed genes was generated per batch selecting those genes with a FC > 2 or FC < 0.5 and the final list was defined as the intersection of the per-batch lists. Heat map plots were generated with the gplots R package.

## Supplementary Information

Supplementary Information.
