# Supplementary material for: Using antisense oligonucleotides for the physiological modulation of the alternative splicing of NF1 exon 23a during PC12 neuronal differentiation
Source: Sci Rep. 2021 Feb 11;11:3661. doi: 10.1038/s41598-021-83152-w (PMC7878752; doi:10.1038/s41598-021-83152-w)
Supplement: Supplementary file 1 — Supplementary Information. [file 41598_2021_83152_MOESM1_ESM.pdf]

Using antisense oligonucleotides for the physiological modulation of the alternative splicing of *NF1* exon 23a during PC12 neuronal differentiation

Josep Biayna[1,#], Helena Mazuelas[1], Bernat Gel[1], Ernest Terribas[1,4], Gabrijela Dumbovic[2], Inma Rosas[1], Juana Fernández-Rodriguez[3,4], Ignacio Blanco[5], Elisabeth Castellanos[1], Meritxell Carrió[1], Conxi Lazaro[3,4], Eduard Serra[1,4*]

[1] Hereditary Cancer Group, Germans Trias i Pujol Research Institute (IGTP), Can Ruti Biomedical Campus, Badalona (Barcelona), Spain.

[2] BioFrontiers Institute, University of Colorado Boulder, CO, USA.

[3] Hereditary Cancer Program, Catalan Institute of Oncology (ICO), Institut d'Investigació Biomédica de Bellvitge (IDIBELL), Hospitalet de Llobregat (Barcelona) Spain..

[4] Centro de Investigación Biomédica en Red de Cáncer (CIBERONC), Spain

[5] Clinical Genetics and Genetic Counseling Program, Germans Trias i Pujol Hospital, Can Ruti Biomedical Campus, Badalona, Barcelona, Spain.

[#] Current address: Institute for Research in Biomedicine (IRB Barcelona), Parc Cientific de Barcelona, Barcelona, Spain (JB)

*To whom correspondence should be addressed.

Eduard Serra; Tel: (+34) 935543067, email: eserra@igtp.cat

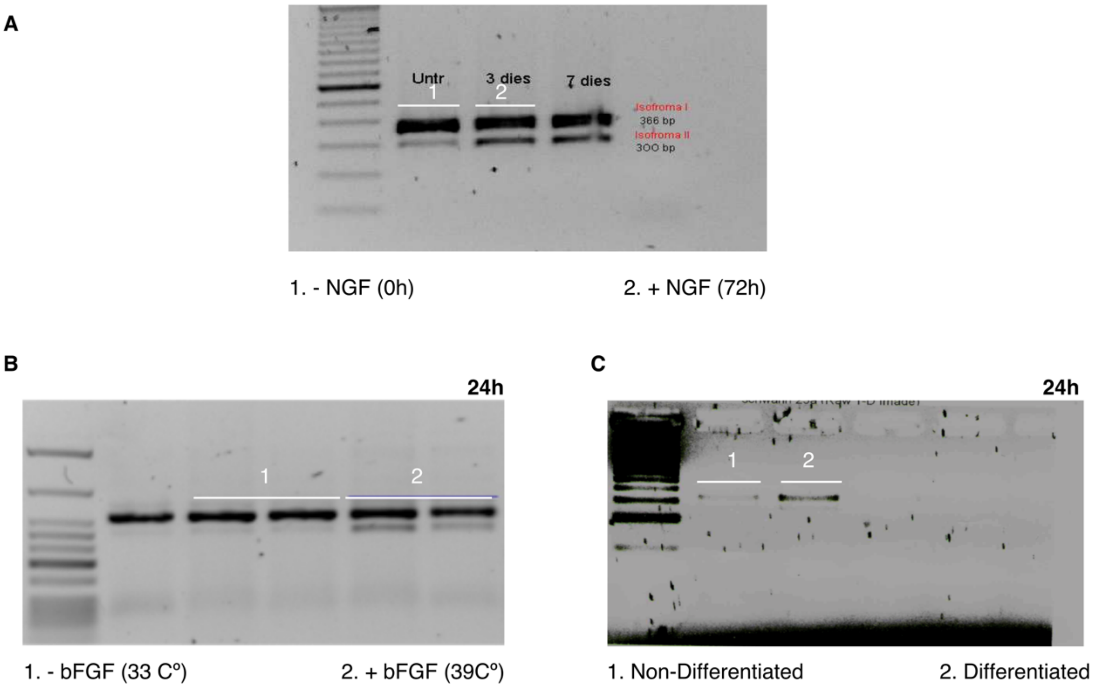

Supplementary Figure S1: Whole agarose gels showing Type II/I isoform expression ratio analysis by RT-PCR supporting Figure 1. **A)** PC12 differentiation, **B)** H19-7/IGFR cells; **C)** Schwan cell differentiation.

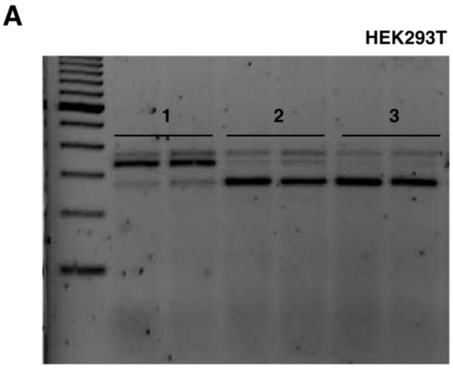

1. PMO_SkpE23a (0uM)
2. PMO_SkpE23a (10uM)
3. PMO_SkpE23a (20uM)

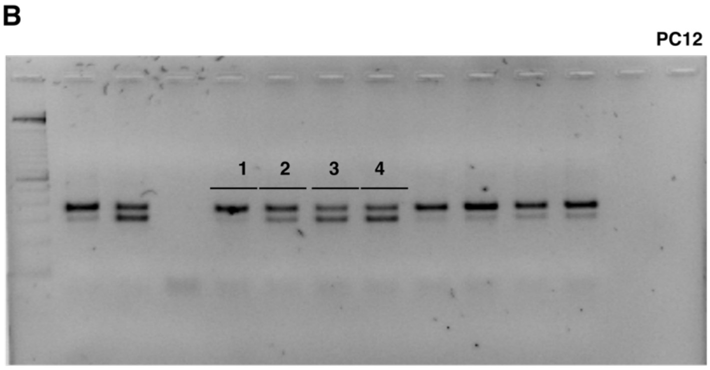

1. PMO_SkpE23a (3h)
2. PMO_SkpE23a (24h)
3. PMO_SkpE23a (48h)
4. PMO_SkpE23a (72h)

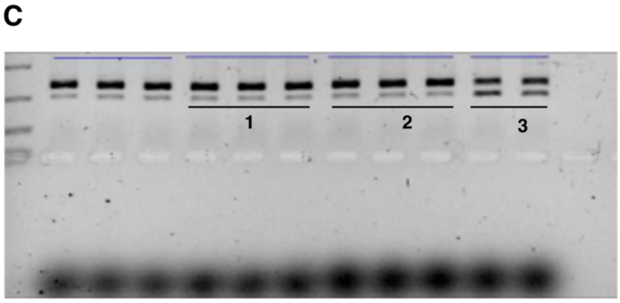

1. TA
2. NTC PMO
3. PMO_SkpE23a

Supplementary Figure S2: Whole agarose gels showing Type II/I isoform expression ratio analysis by RT-PCR supporting Figure 2. The effect of PMO treatment on E23a splicing as well as specificity is measured. Different cell types were used: HEK293T **(A)** and PC12 cells **(B, C)**.

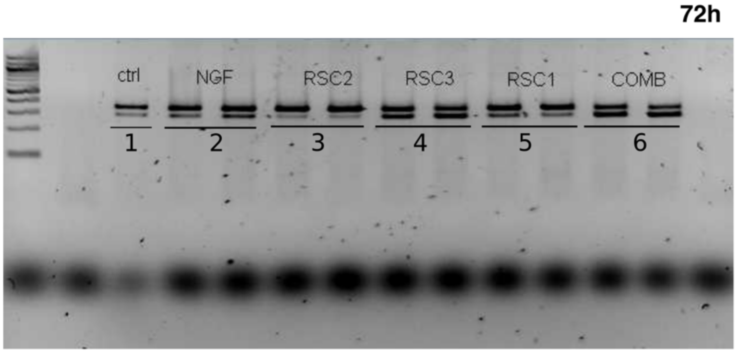

1. TA (-NGF)
2. + NGF
3. PMO_CSRII
4. PMO_CSRIII
5. PMO_ CSRI
6. PMO_All (CSR I/II/III)

Supplementary Figure S3: Whole agarose gel showing Type II/I isoform expression ratio analysis by RT-PCR of the PMO-IncE23a design supporting Figure 3.

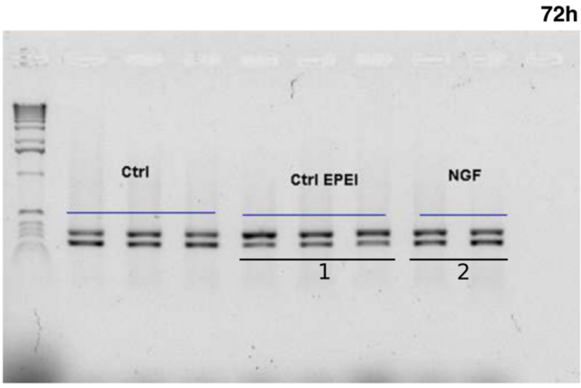

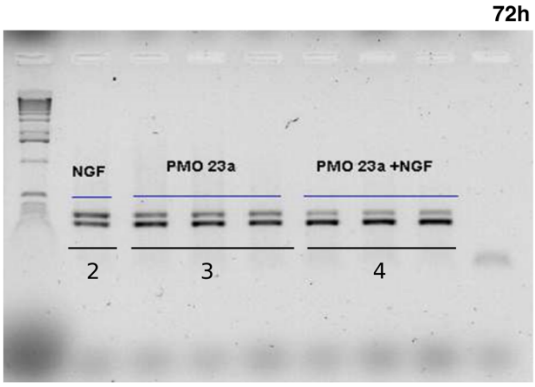

1. TA (-NGF)
2. + NGF
3. PMO_SkpE23a
4. PMO_SkpE23a + NGF

Supplementary Figure S4: Whole agarose gels of Type II/I isoform expression ratio analysis by RT-PCR, supporting Figure 4.

A

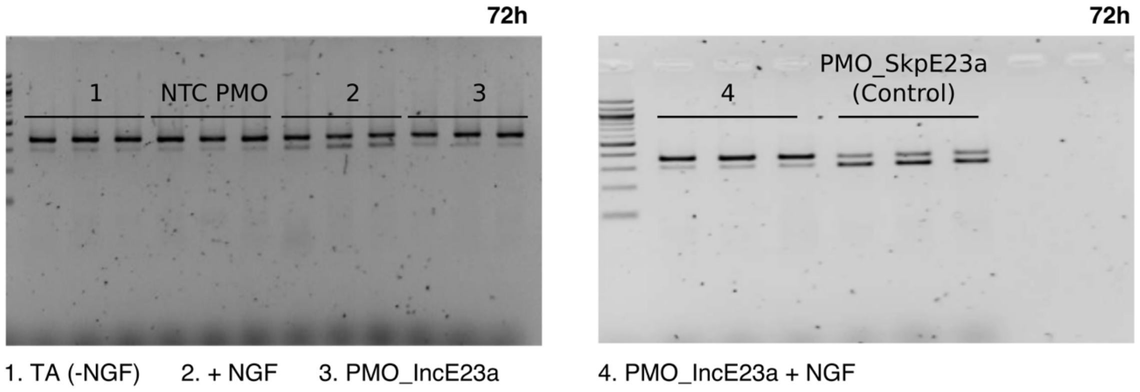

B

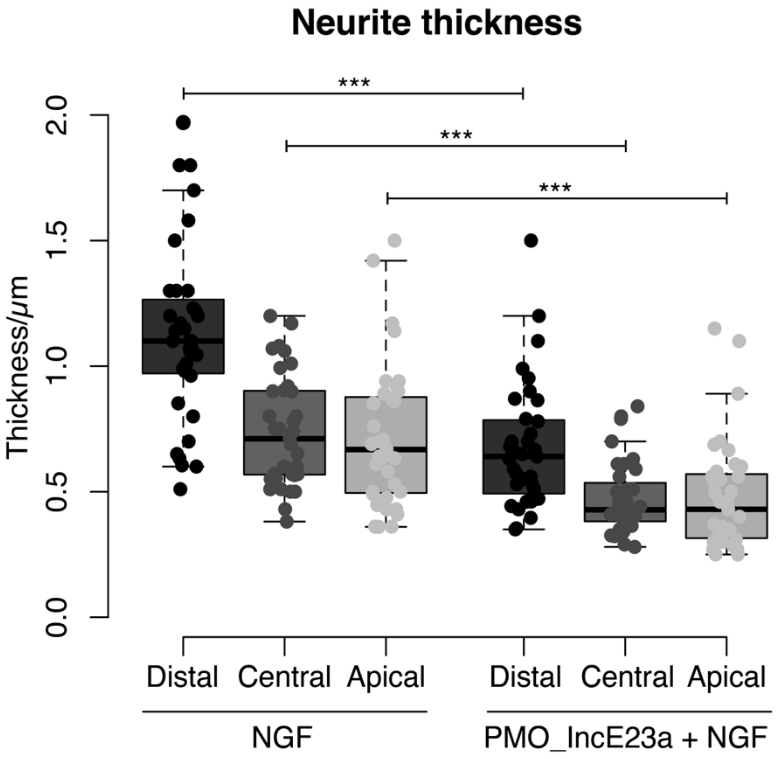

Supplementary Figure S5: A) Whole agarose gels showing Type II/I isoform expression ratio analysis by RT-PCR, supporting Figure 5. TA: transfection agent, NTC: Non-targeting control. **B)** Neurite thickness (µm) at different neurite location of the cell body (distal, central and apical) for cells treated with NGF and NGF + PMO_IncE23a.

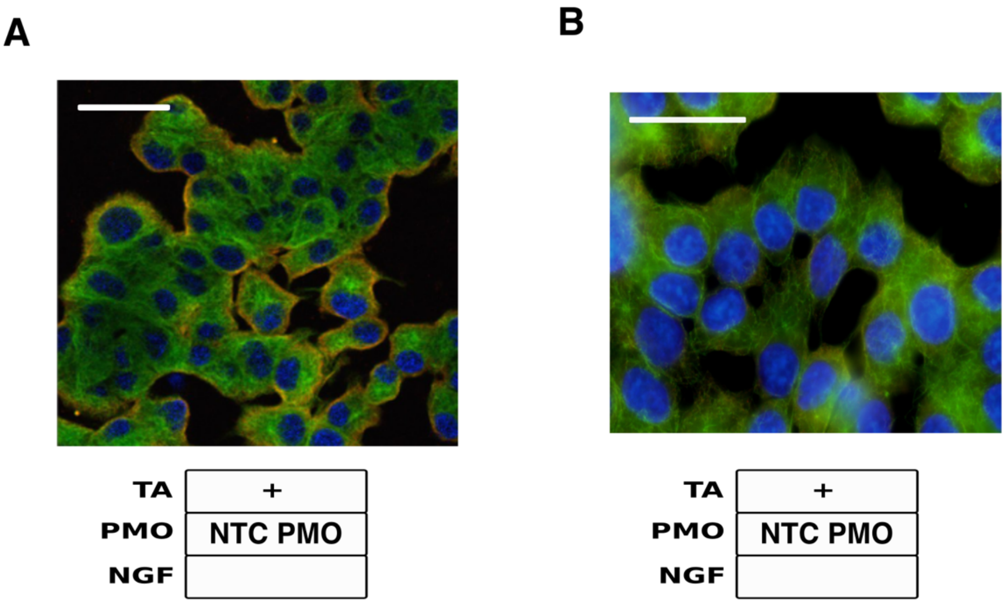

Supplementary Figure S6: Representative images of double immunofluorescent (IF) staining of NGFR (red) and α-tubulin (green) of PC12 cells after 72h. Nucleus stained with DAPI (blue). Experimental conditions are summarized in a box below each image. TA: transfection agent, NTC: Non-targeting control. **A**) NTC control for experiment shown in figure 4. Scale bar, 40 µm. **B**) NTC control for experiment shown in figure 5. Scale bar, 30 µm.

P-ERK 1/2 levels (72h)

PMO-SkpE23a

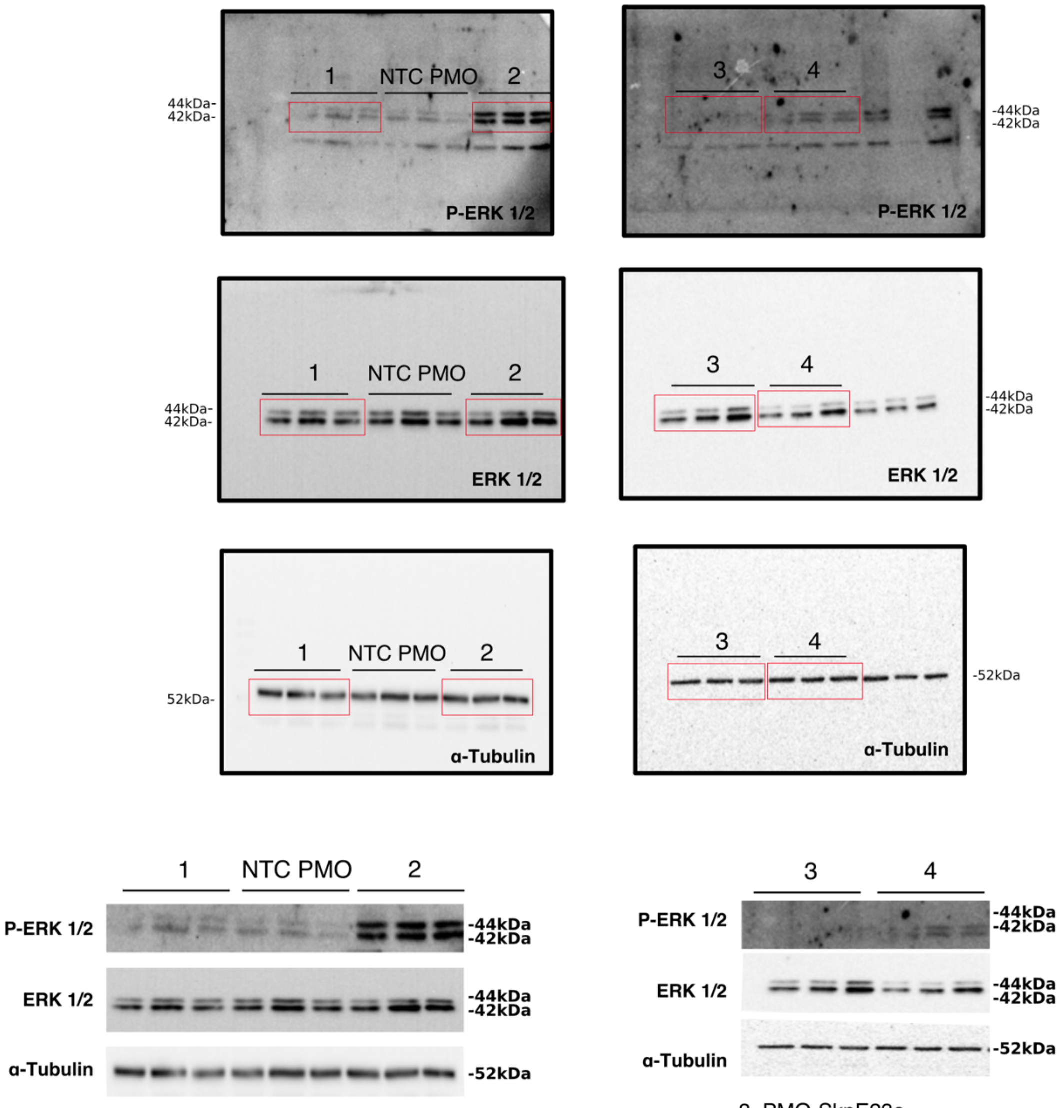

1. TA (-NGF)
2. + NGF

3. PMO-SkpE23a
4. PMO-SkpE23a + NGF

P-ERK 1/2 levels (72h)

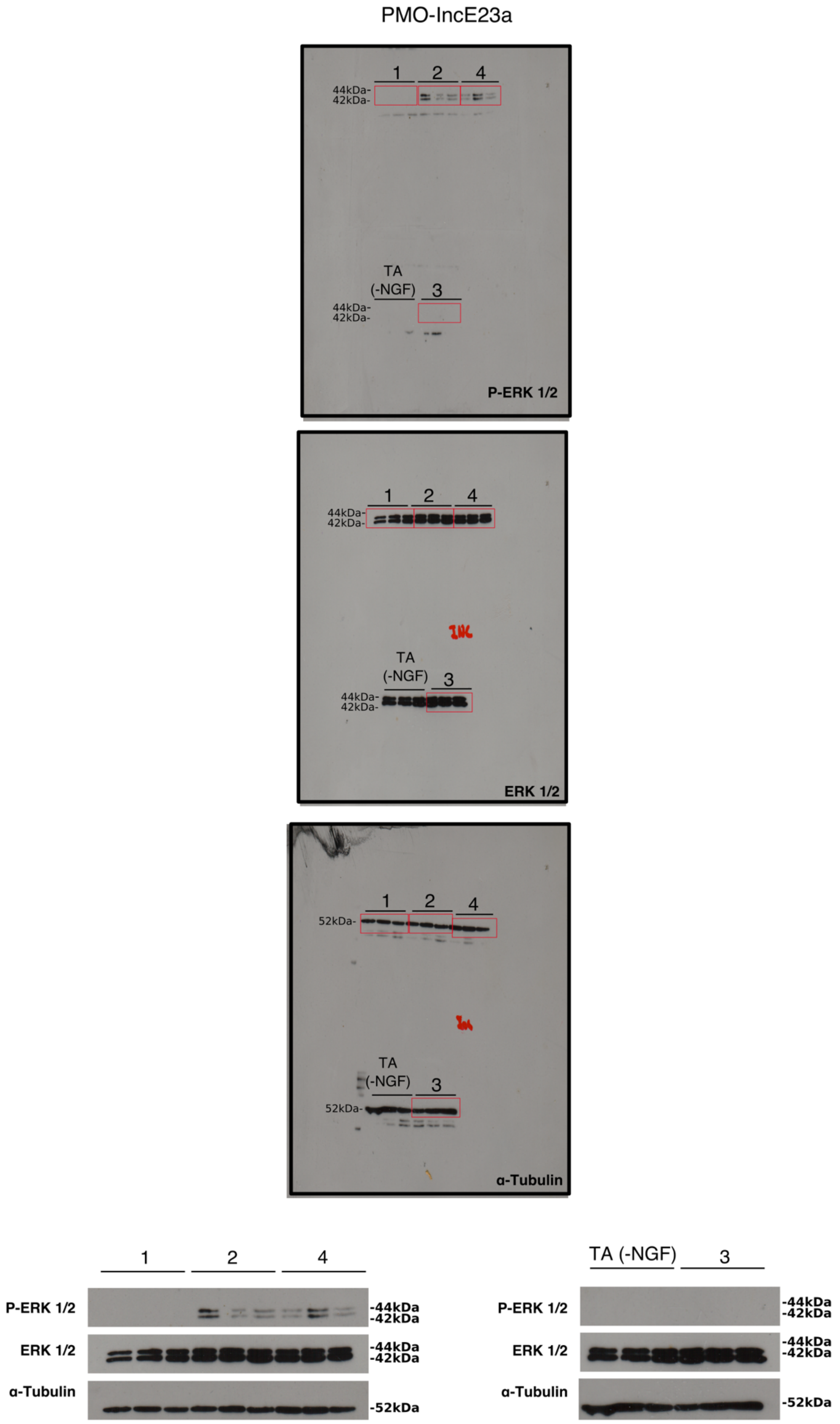

1. TA (-NGF)
2. TA+NGF
4. PMO-lncE23a + NGF

3. PMO-lncE23a

P-PKA α/β levels (48h)

PMO-SkpE23a

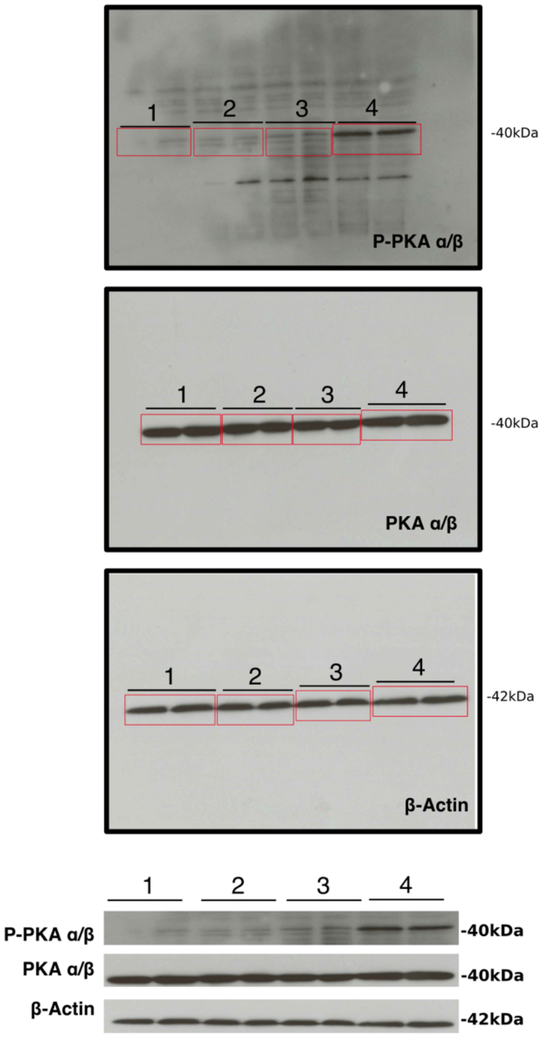

1. TA (-NGF)
2. + NGF
3. PMO_SkpE23a
4. PMO_SkpE23a + NGF

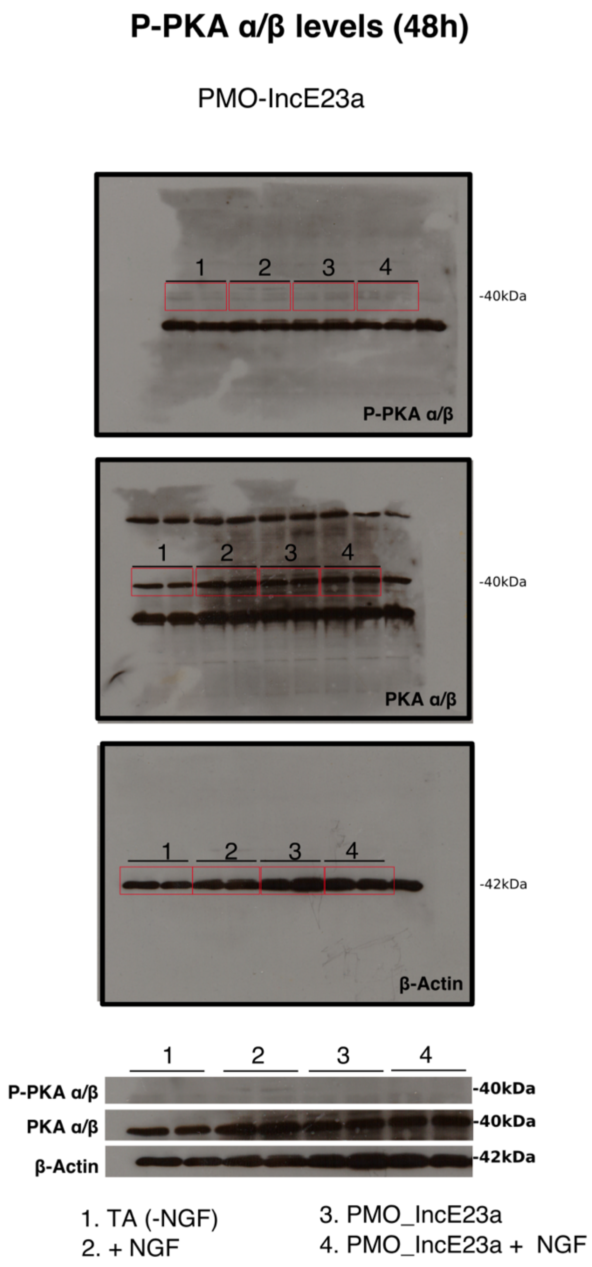

Supplementary Figure S7: Uncropped western blots showing the effect of PMOs on ERK1/2 (42-44 kDa) activation, supporting Figure 6A, and PKA activation, supporting Figure 6B**.** Numbers and red boxes denote the regions of the blot used in the composed Figure 5. TA: transfection agent, NTC: Non-targeting Control.

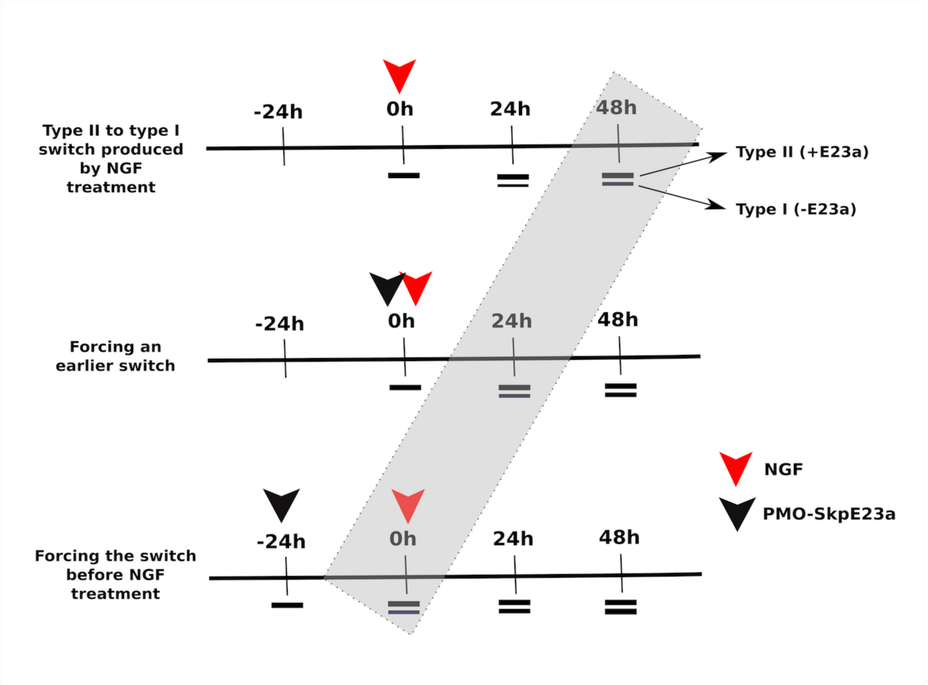

Supplementary Figure S8: Schematic representation of different states of the type II/I switch at the moment NGF is added to PC12 cells. The type II/I switch capacity of NGF (red arrow) over time is shown on the top timeline. The switch is evident at 24h and is highest at 48h. If NGF is added at the same time as PMO-SkpE23a (black arrow), the type II/I proportion induced by NGF alone at 48h, anticipates 24h, as depicted in the middle timeline. When PMO-SkpE23a is added 24h before NGF, bottom timeline, the type II/I proportion induced by NGF alone at 48h is already reached when NGF is added to the PC12 cell culture. This is how the time-dependent effect of type II/I switch was studied using PMOs.

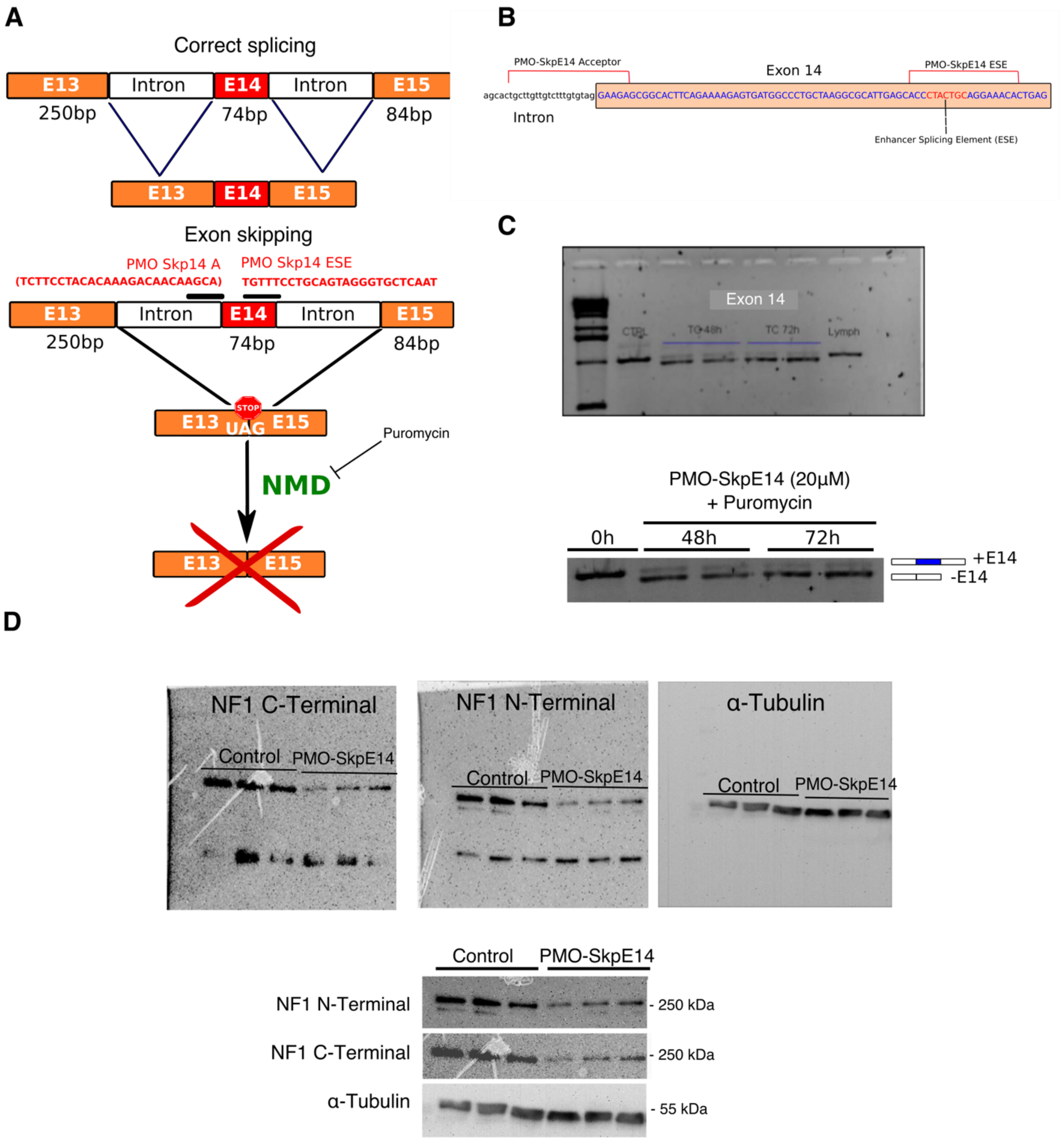

Supplementary Figure S9: Design of a PMO to force E14 skipping and set up of experimental conditions. **A)** Schematic representation of E14 skipping strategy using two specific PMOs (in red). The skipping of E14 produces a shift in the reading frame, generating a *Nf1* mRNA with a premature stop-codon that is degraded by the nonsense mediated decay (NMD) mechanism, unless puromycin is added. **B)** Specific sequence recognition of the two E14 PMOs designed. PMO-SkpeE14 targeting the acceptor site region and PMO-SkpE14 ESE recognizing the Enhance Splicing Element (ESE) are highlighted. For simplicity, the combined treatment of these two PMOs was referred as PMO-SkpE14. **C)** Effect of 20μM PMO-SkpE14 on E14 skipping in PC12 cells at 24h (top) and at 48h and 72h (bottom) analyzed by RT-PCR. To evaluate the degree of E14 skipping, puromycin was used to avoid NMD. **D)** Detection of neurofibromin abundance by western blot. In this case, puromycin was not used to observe the impact of NMD on the final translated neurofibromin.

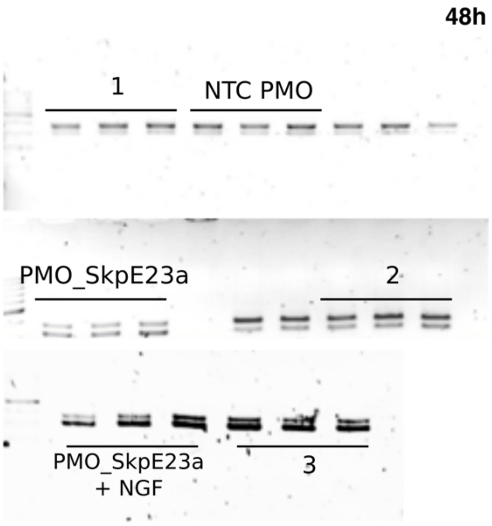

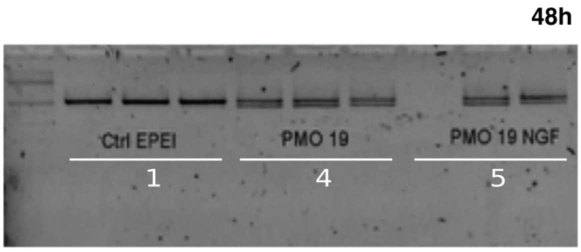

1. TA (-NGF)
2. + NGF
3. PMO_SkpE23a(-24h)+ NGF
4. PMO_SkpE14
5. PMO_SkpE14 + NGF

Supplementary Figure S10: Whole gels of Type II/I isoform expression ratio analysis (left) and exon 14 skipping levels (right) by RT-PCR, supporting Figure 7.

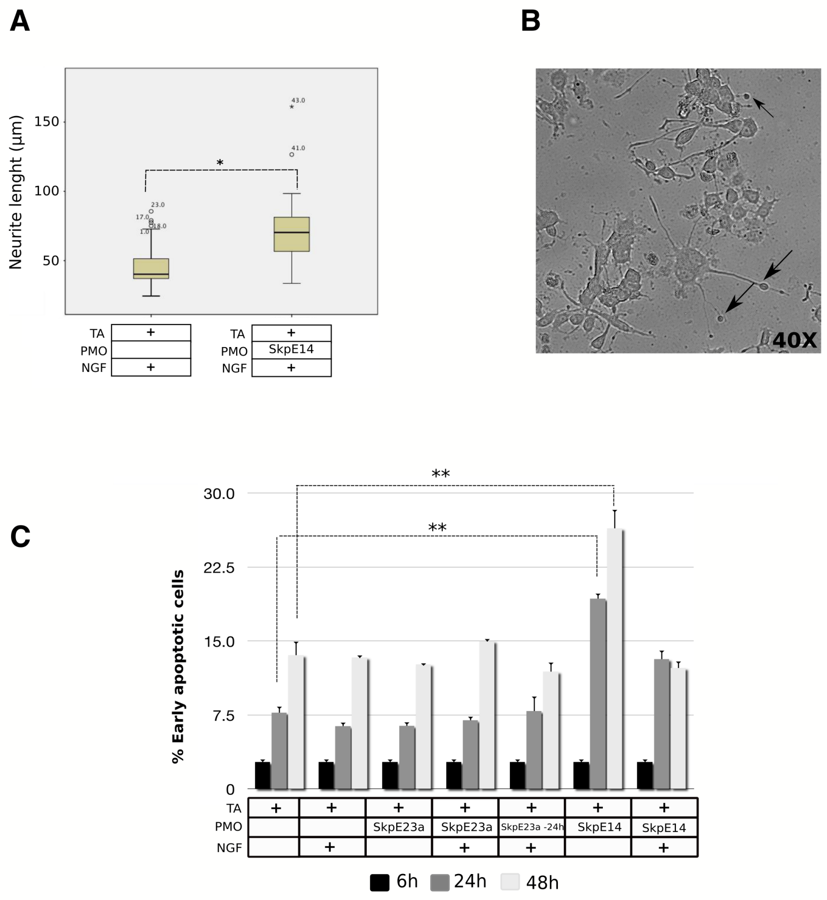

Supplementary Figure S11: Morphological and physiological effects of PMO-SkpE14 on PC12 differentiation. A) Quantification of neurite length (µm) in PC12 cells treated with NGF alone or with NGF plus PMO-SkpE14. **B)** Representative image of PC12 cells treated with NGF and PMO-SkpE14. Black arrows indicate the presence of varicosities in PC12 cells differentiating under these conditions. **C)** Time-course analysis of early apoptotic cells under the different experimental conditions depicted in the boxes below the graphs. For all the graphs, error bars indicate ±SEM. * $P < 0.05$, ** $P < 0.01$ as evaluated by paired *t*-test vs control cells. TA: transfection agent.

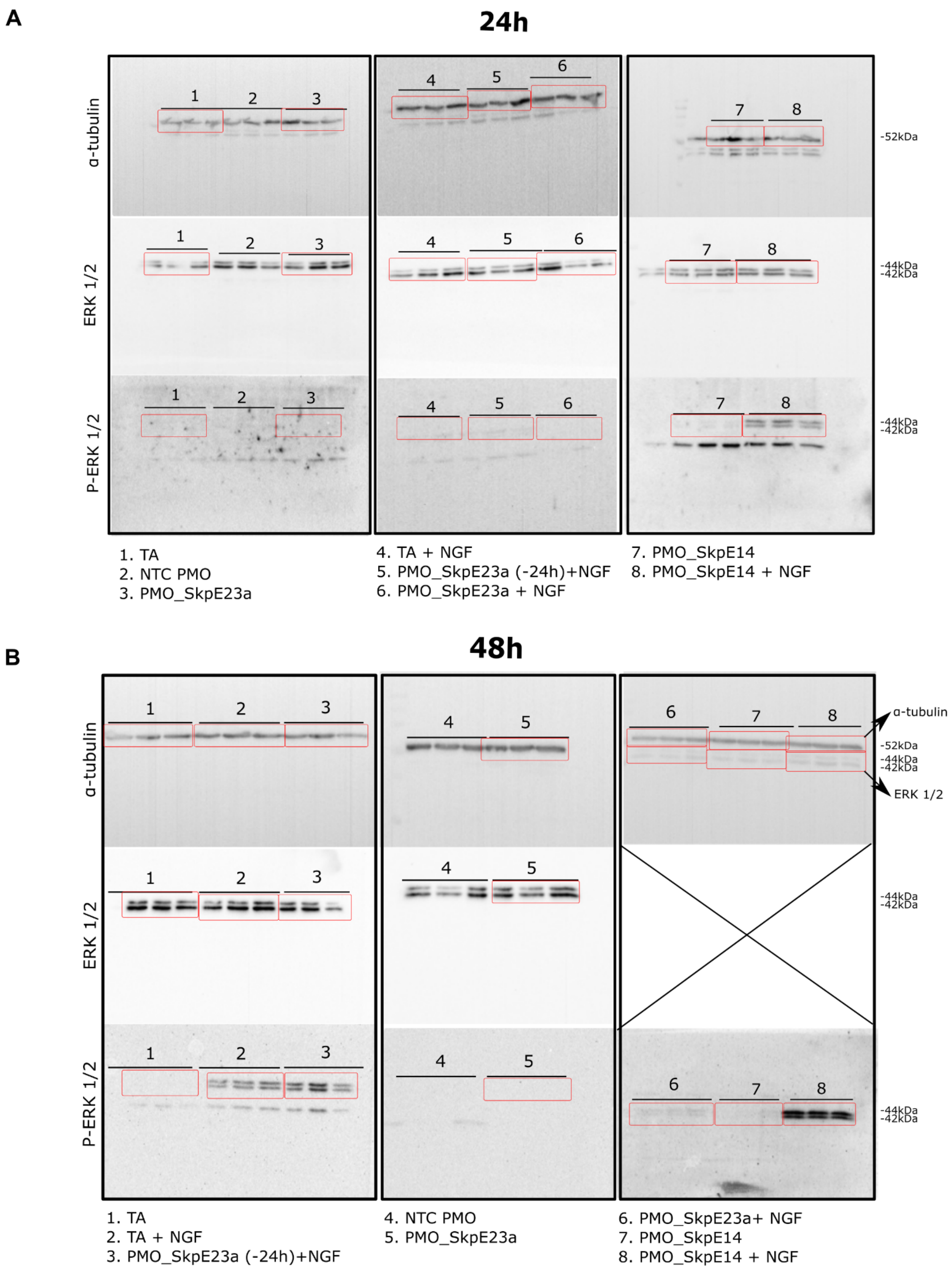
A
24h
α-tubulin
ERK 1/2
P-ERK 1/2
1 2 3 4 5 6 7 8
-52kDa
-44kDa
-42kDa
1. TA
2. NTC PMO
3. PMO_SkpE23a
4. TA + NGF
5. PMO_SkpE23a (-24h)+NGF
6. PMO_SkpE23a + NGF
7. PMO_SkpE14
8. PMO_SkpE14 + NGF
B
48h
α-tubulin
ERK 1/2
P-ERK 1/2
1 2 3 4 5 6 7 8
-52kDa
-44kDa
-42kDa
1. TA
2. TA + NGF
3. PMO_SkpE23a (-24h)+NGF
4. NTC PMO
5. PMO_SkpE23a
6. PMO_SkpE23a+ NGF
7. PMO_SkpE14
8. PMO_SkpE14 + NGF

C

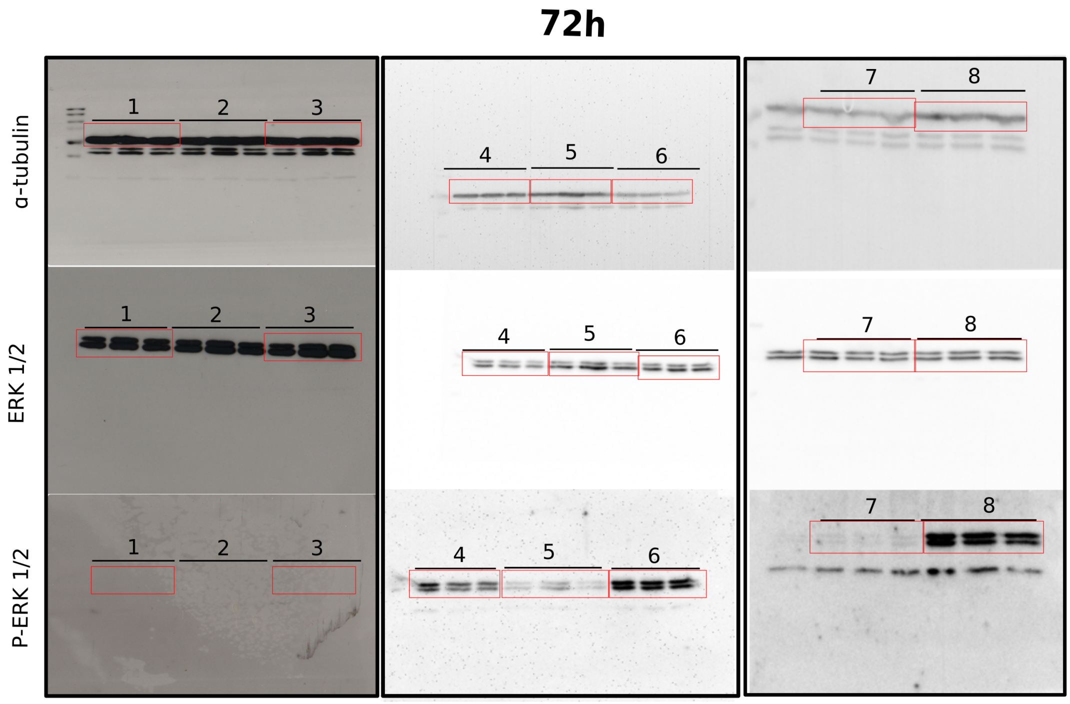

1. TA
2. NTC PMO
3. PMO_SkpE23a

4. TA + NGF
5. PMO_SkpE23a + NGF
6. PMO_SkpE23a (-24h) + NGF

7. PMO_SkpE14
8. PMO_SkpE14 + NGF

D

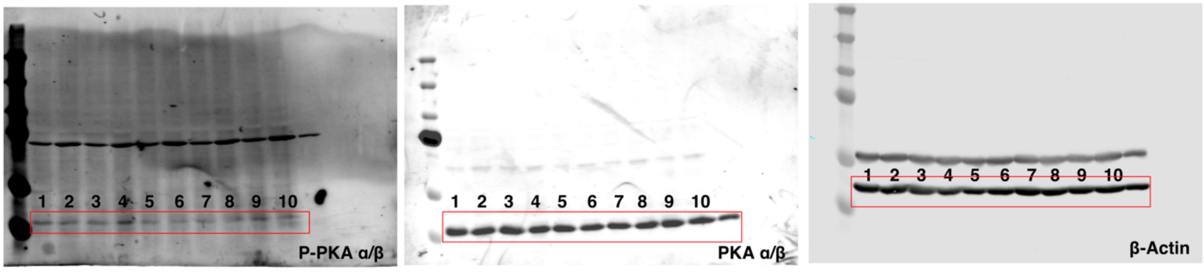

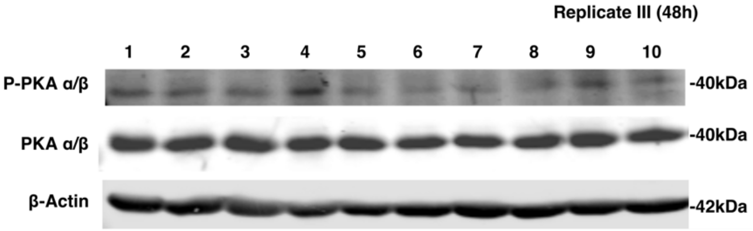

1. TA
2. TA + NGF
3. PMO_SkpE23a
4. PMO_SkpE23a + NGF
5. PMO_SkpE23a (-24h)
6. PMO_SkpE23a (-24h) + NGF
7. PMO_IncE23a
8. PMO_IncE23a + NGF
9. PMO_SkpE14
10. PMO_SkpE14 + NGF

Supplementary Figure S12: Whole western blots analyzing the dynamic use of PMOs on ERK1/2 (42-4 kDa) activation, supporting Figure 8A, and PKA activation, supporting Figure 8B. **A)** ERK1/2 activation at 24h. **B)** ERK1/2 activation at 48h. **C)** ERK1/2 activation at 72h. Numbers and red boxes denote the regions of the blot used for quantification of Figure 8A. **D)** Western blot analysis of P-PKA (40 kDa) and total PKA levels for the different experimental conditions at 48h, in a third replicate and as part of Figure 8B quantifications. TA: transfection agent, NTC: Non-targeting Control.

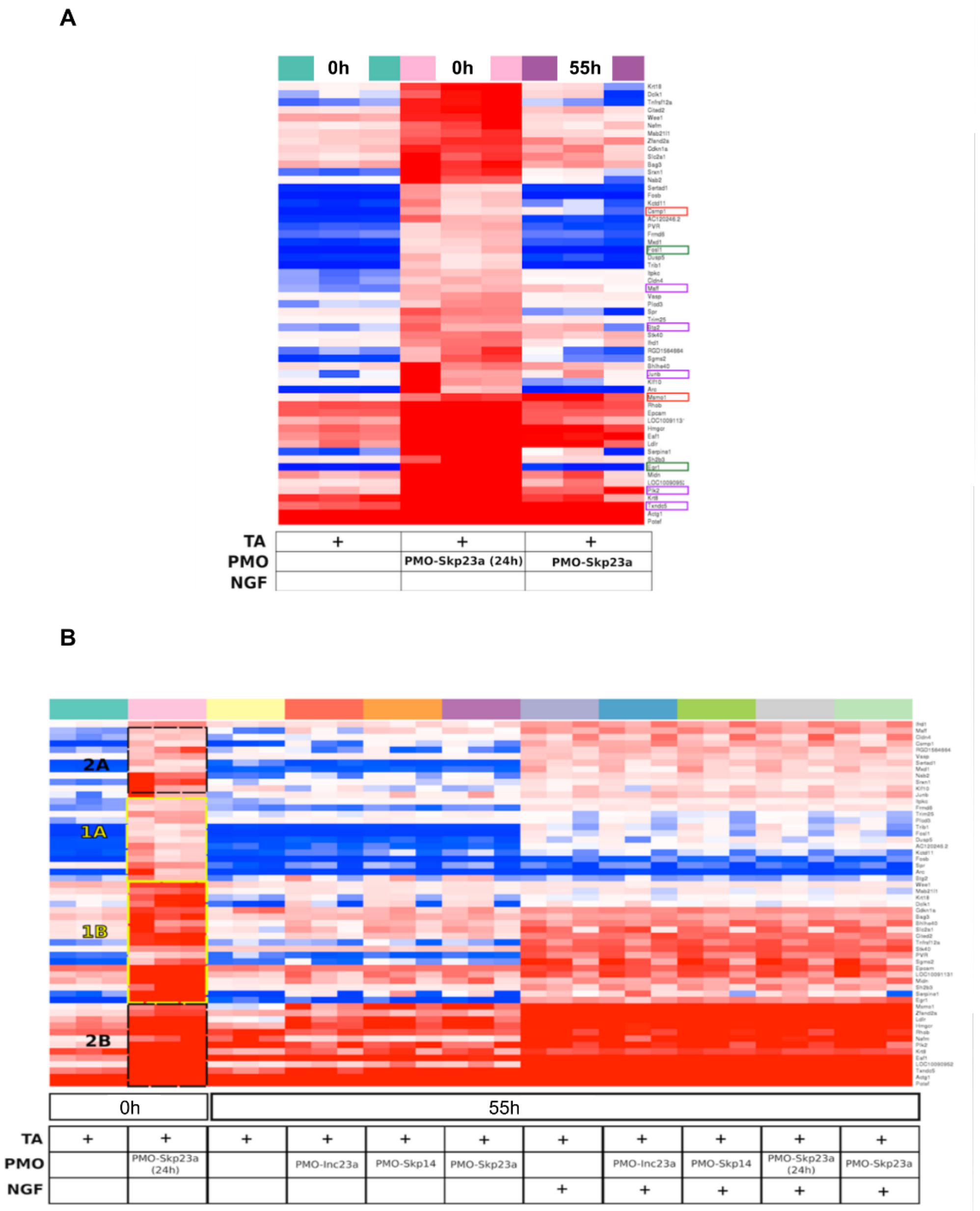

Supplementary Figure S13: A) Heatmap of differentially expressed genes with a fold change greater than 2 (FC>2) at different experimental conditions depicted in the boxes below. Note than at the time PC12 cells were treated with NGF (0h) there were many NGF-activated genes already upregulated in PC12 cells pre-treated for 24h with PMO-Skp-E23a (Supplementary Table S3). 55h after the addition of NGF, the same cells recovered the expression patterns of control PC12 cells only treated with transfection agent despite the still active capacity of PMO-Skp-E23a to force the type II/I switch. Known NGF-response genes are highlighted. **B)** Heatmap of differentially expressed (FC>2) genes in a broader range of conditions depicted in the boxes below. Clusters of genes activated in conditions in which PC12 cells were treated for 24h with PMO-SkpE23a are highlighted and summarized in Supplementary Table S3.

PMO	Sequence 5'-3'	Target Position/Exon
PMO-SkpE23a	GTTGCCTACAGAACAGAGATGAGCA	**Exon 23a**
PMO-IncE23a	AAAAACAATGTTGGCTTGTACATAA	**Intron-Exon 23a**
PMO-SkpE14	TCTTCCTACACAAAGACAACAAGCA	**Exon 14 Acceptor**
PMO-SkpE14	TGTTTCCTGCAGTAGGGTGCTCAAT	**Exon 14 ESE**
PMO-CSRI	GTAACACAAATGGAACCAACAGAAA	**Intron-Exon 23a**
PMO-CSRIII	CACAGCACCGACTACATACAGCAAC	**Intron-Exon 23a**

Supplementary Table S1. List of PMOs used in this study

Primer Name	Sequence 5' – 3'	Species
**Control E3 Forward**	CAGAACACACATACCAAAGTCA	Rat
**Control E3 Reverse**	GACCAGCATTGTTTCATCTA	Rat
**Forward E23-2**	CAGAGTTCCCCTCGCAGCTTCG	Rat
**Reverse E25**	CTCCGTGCCAAGTCGGAGTTGC	Rat
**Forward E23a**	TTAGAACCATCAGAGAGCC	Human
**Reverse E23a**	TTTCGATTCTAGGTGGTG	Human
**Forward E14**	CAGGCAGATAGAAGTTCCTGTCA	Rat
**Reverse E14**	GTTTTGTAGCCTGTTCCCACTTTGC	Rat
**Forward E14**	GCAGGCAGATAGAAGTTCCTGTCAC	Human
**Reverse E14**	GTTCTACAAATTGAGTATTGGTATCAG	Human
**Forward E23-2/E24**	GTTTGTACCAGGTGGTTAGC	Rat
**Forward E23-2/E23a**	TTTGTACCAGGCAACTTG	Rat
***Gap43* Forward**	CCGACAGGATGAGGGTAAAG	Rat
***Gap43* Reverse**	GCAGGAGAGACAGGGTTC	Rat
***Mmp3* Forward**	TGAAGATGACAGGGAAGCTGG	Rat
***Mmp3* Reverse**	GGCTTGTGCATCAGCTCCAT	Rat
***Dusp6* Forward**	TCTTTGGCTCCACTATACGCAA	Rat
***Dusp6* Reverse**	ATCCAGGCAATAGGTTTGCTTC	Rat
***Mbnl1* Forward**	ATGGCTGTTAGTGTCACACCA	Rat
***Mbnl1* Reverse**	CATGTTCTTCTGCTGAATCAA	Rat
***Mbnl2* Forward**	CAGGTTGAAAATGGAAGAGTAA	Rat
***Mbnl2* Reverse**	TTGAGCCCGGGACAGTGACCGG	Rat
***Celf3* Forward**	GACCGGAAGCTCTTTGTGGGG	Rat
***Celf3* Reverse**	AGAGTCCGGCTGCTGTGA	Rat
***Rpl19* Forward**	ATCGCCAATGCCAACTCT	Rat
***Rpl19* Reverse**	GAGAATCCGCTTGTTTTTGAA	Rat
***Rpl29* Forward**	ACAGAAATGGCATCAAGAAACCC	Rat
***Rpl29* Reverse**	TCTTGTTGTGCTTCTTGGCAA	Rat

Supplementary Table S2. List of primer sequences used in this study

A

Genes
Vasp
Fosb
PVR
Itpkc
Sertad1
Wee1
Bag3
Fosl1
Dusp5
Hmgcr
Plk2
Dclk1
Mab21l1
Sgms2
Spr
Mxd1
Bhlhe40
Potef
RGD1564664
Srxn1
Slc2a1
Stk40
LOC100911319
Epcam
Rhob
Ifrd1
Frmd6
Midn
Nab2
Klf10
Trib1
Arc
Maf f
Krt8
Krt18
Ldlr
Csrnp1
Btg2
Tnfrsf12a
Kctd11
Trim25
Plcd3
Actg1
Nefm
Txndc5
Eaf1
Msmo1
Egr1
AC120246.2
Junb
Cdkn1a
Zfand2a
Serpine1
LOC100909521
Cldn4
Sh2b3

B

**Cluster 1A**	Kif4a, Brca1, Pole, Ncaph, Slfn13, Chaf1a, Cit, Espl1, Dtl, Esco2, Kif18b, Mybl2, Rrm[illegible] Mcm3, Tk1, Plxna4a, Abca1, Tspan18
**Cluster 1B**	Arhgap11a, Bub1, Mcm6, Kif11, Prc1, LOC10035953, Mcm5, Kif22, Spag5, **Tpx2**, **Tacc3**, **Iqgap3**
**Cluster 2A**	Stmn2, Ampd3, Cyp51, Ptp4a1, Cd63, Fdps, Msmo1, Zfand2a, Sdcbp, Slc6a8, Btg1, Sa[illegible] Gapdh-ps2, Hmgcr, Adm, Ldlr, Sdc1, Fbxo32
**Cluster 2B**	Tfrc, Atp11a, Pgam1, Mmd, Eml5, Eaf1, Pgk1

Supplementary Table S3. A) List of differentially expressed genes (fold change > 2) shown in Supplementary Figure S9A. **B)** List of differentially expressed genes (fold change > 2) for each of the colored clusters of Supplementary Figure S9B. Known genes related with neurite outgrowth and formation are marked in bold.
